# Molecular Mechanism of Lipid Nanodisk Formation by Styrene-Maleic Acid Copolymers

**DOI:** 10.1016/j.bpj.2018.06.018

**Published:** 2018-06-20

**Authors:** Minmin Xue, Lisheng Cheng, Ignacio Faustino, Wanlin Guo, Siewert J. Marrink

**Affiliations:** 1 State Key Laboratory of Mechanics and Control of Mechanical Structures, Key Laboratory for Intelligent Nano Materials and Devices of the Ministry of Education, Institute of Nanoscience, Nanjing University of Aeronautics and Astronautics, Nanjing, People’s Republic of China; 2 Groningen Biomolecular Science and Biotechnology Institute, University of Groningen, Groningen, the Netherlands; 3 Zernike Institute for Advanced Materials, University of Groningen, Groningen, the Netherlands; 4 College of Mechanical and Electrical Engineering, Beijing University of Chemical Technology, Beijing, People’s Republic of China

## Abstract

Experimental characterization of membrane proteins often requires solubilization. A recent approach is to use styrene-maleic acid (SMA) copolymers to isolate membrane proteins in nanometer-sized membrane disks, or so-called SMA lipid particles (SMALPs). The approach has the advantage of allowing direct extraction of proteins, keeping their native lipid environment. Despite the growing popularity of using SMALPs, the molecular mechanism behind the process remains poorly understood. Here, we unravel the molecular details of the nanodisk formation by using coarse-grained molecular dynamics simulations. We show how SMA copolymers bind to the lipid bilayer interface, driven by the hydrophobic effect. Due to the concerted action of multiple adsorbed copolymers, large membrane defects appear, including small, water-filled pores. The copolymers can stabilize the rim of these pores, leading to pore growth and membrane disruption. Although complete solubilization is not seen on the timescale of our simulations, self-assembly experiments show that small nanodisks are the thermodynamically preferred end state. Our findings shed light on the mechanism of SMALP formation and on their molecular structure. This can be an important step toward the design of optimized extraction tools for membrane protein research.

## Introduction

Membrane proteins are of great importance to a variety of essential physiological functions in all organisms. Encoded by 30% of all genes, membrane proteins account for almost 70% of known drug targets in the cell. However, they only contribute less than 2% of the structures in the Protein Data Bank (1). These proteins are relatively less studied because of a lack of experimental approaches. One of the major challenges in membrane protein research is the isolation of these proteins without destroying their stability and activity. Extraction of membrane proteins from their lipid environments can lead to their inactivation or aggregation.

A widely used solution is to incorporate the protein into a model lipid membrane. In particular, lipid nanodisks have proven to be an efficient way to solubilize membrane proteins while keeping a natural environment (2, 3, 4, 5, 6). In the pioneering work of Sligar and co-workers, these small bilayer patches are surrounded and stabilized by a ring of *α*-helical peptides (also called membrane scaffold proteins (MSP)) (7, 8). One disadvantage is that in preparing these MSP nanodisks, one relies on the use of surfactants hindering the study of membrane proteins in their native lipid environment. Besides, the use of peptides as rim-stabilizing molecules complicates the use of biophysical techniques such as circular dichroism, Fourier transform infrared and NMR spectroscopies (9).

An alternative approach to MSP is the use of amphipathic copolymers. These copolymers keep membrane proteins soluble without detergents (9, 10, 11, 12, 13, 14). This implies that membrane proteins, together with their annular lipid shells, can be extracted directly from native cellular membranes or from reconstituted vesicles. An efficient copolymer introduced by Dafforn and coworkers (9, 10) is composed of styrene-maleic acid (SMA) units. SMA molecules, together with lipids, spontaneously form disk-shaped particles of 10–12 nm in diameter, which are denoted as SMA lipid particles (SMALPs) (15). Bigger particles may also form depending on the shape and diameter of the embedded protein(s), polymer composition, and the polymer/lipid ratio (10, 16, 17, 18, 19). Importantly, SMA copolymers dissolve in a wide range of membranes without showing specificity for any lipid types (20, 21, 22). They have been used to characterize the annular lipid shells of a variety of membrane proteins (23, 24, 25). The intrinsic hydrophobicity (SMA ratio) and the protonation state of maleic acid groups strongly influence the rate of membrane solubilization (26, 27). These properties, together with the varying molecular weight, make SMA copolymers easy to change and adjust (28, 29). As a result, these pH-responsive copolymers have also been used as membrane-destabilizing polymers for the delivery of therapeutic molecules (26, 30).

Despite the promising future of SMA copolymers in membrane protein research, little is known about the molecular mechanism of SMA-lipid nanodisk formation. Scheidelaar and colleagues suggested a model for membrane solubilization by SMA copolymers in which the hydrophobic effect would drive the interaction between SMA copolymers and membranes, modulated by electrostatic interactions (20). Molecular dynamics (MD) simulations provide an attractive tool to study the molecular interactions and the dynamics of the solubilization process in detail (31). Considering the large molecular weight of the polymers, coarse-grained (CG) models are required to access the relatively large timescales involved in membrane destabilization (32, 33, 34, 35). A CG model that has been parametrized for both polymeric systems and lipid membranes is the Martini model (36). This model has already been successfully applied to simulate the interaction of a variety of polymers and lipid membranes, including studies on polymer adsorption (37, 38, 39, 40, 41, 42, 43), polymer-mediated fusion (44), the permeation process of dendrimers (45) and polymer-coated nanoparticles (46, 47), and preformed lipid nanodisks (48, 49, 50, 51, 52, 53).

Here, based on CG MD simulations with the Martini model, we describe the molecular mechanism of action of SMA copolymers in destabilizing a model lipid bilayer. We provide detailed insight into the insertion, penetration, and pore formation of these copolymers and show how they cooperatively lead to complete destabilization of the lipid membrane and the onset of nanodisk formation. Besides, self-assembly experiments of SMA copolymers and lipids of different length show that small nanodisks are the preferred end state.

## Methods

### CG SMA model

**Figure 1:**
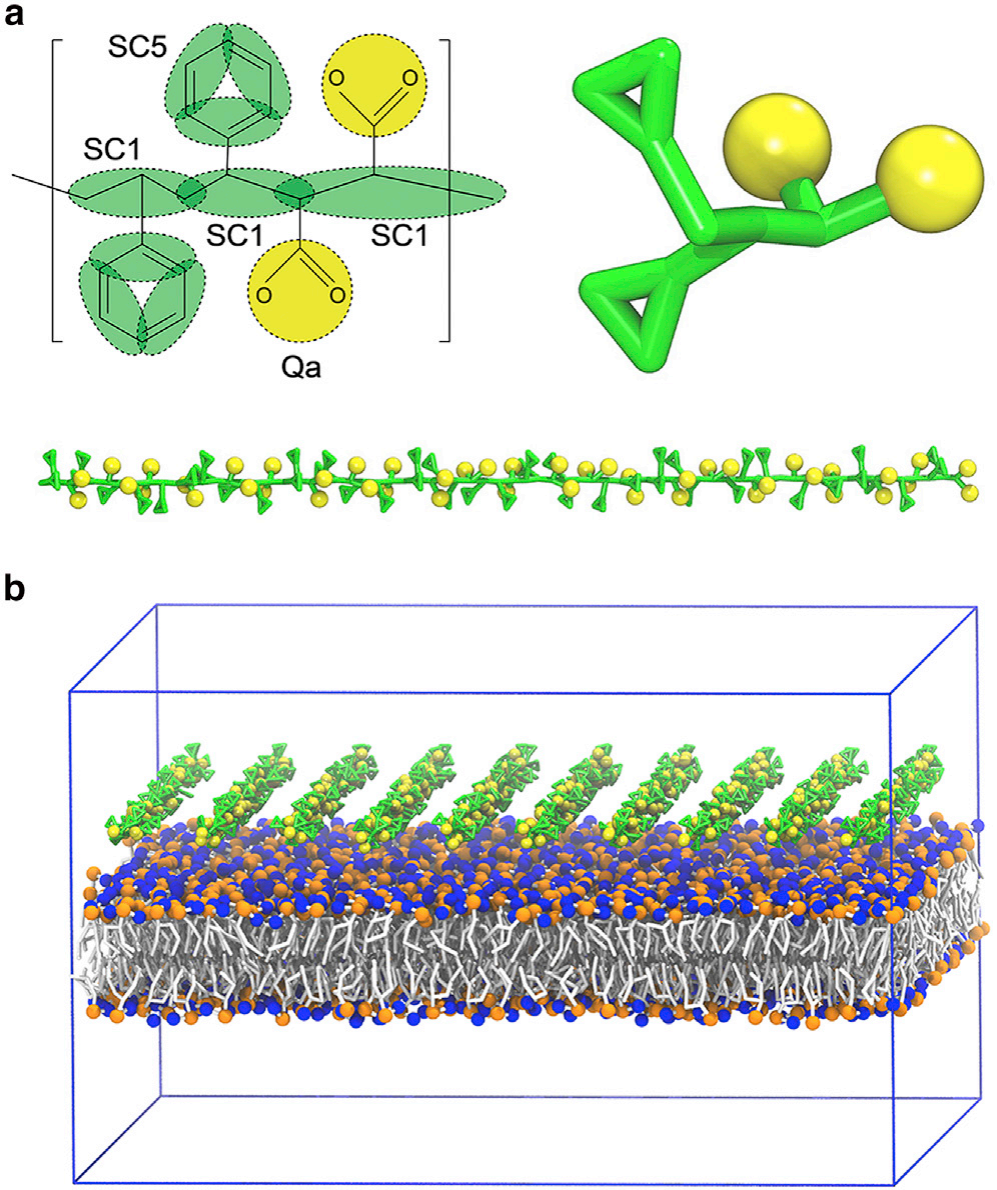
SMA model and starting configuration of the simulation. (*a*) A CG model for the SMA copolymer with mapping of the CG SMA model and chosen bead types is shown at the top left, and a zoomed-in image of one CG unit is shown at the top right. (*b*) The initial configuration of the simulation system with 10 SMA copolymers above the preformed DDPC lipid bilayer is shown. DDPC lipids are shown in gray with phosphate groups in orange and choline groups in blue. The SMA copolymers are shown in green, and the carboxyl groups are shown in yellow. The periodic boundary condition box is shown with blue solid lines. The solvent is omitted for clarity. To see this figure in color, go online.

The Martini CG model is used for the parametrization of the basic SMA units (54). Herein, we used SMA copolymers consisting of 23 units, with each unit including two styrene groups and one maleic acid, yielding a molecular weight of ∼7.4 kDa, which is similar to the molecular weight used in previous experiments (9, 13, 27). The copolymers were treated as fully deprotonated with two negative charges in each repeating unit to obtain the high aqueous solubility of these copolymers and to avoid aggregation. For the styrene group, a three-bead mapping scheme was used, similar to the ring-based side chains in the existing Martini models for the aromatic phenylalanine and tyrosine amino acids and the styrene group in the polystyrene molecule (55, 56). For the maleic acid groups, a one-bead representation was used to represent the carboxylic group, carrying a full negative charge each. The chosen mapping of the CG SMA copolymer is shown in Fig. 1 *a*.

Bonds and improper dihedral angles were represented based on standard harmonic potentials, whereas angles and proper dihedral angles were modeled with cosine-based potentials and periodic dihedral potentials, respectively. The set of CG bonded parameters was parametrized by comparison with atomistic simulations of the SMA copolymers at the interface between an aqueous solution and dodecane. Constraints were applied to the aromatic CG beads instead of using ordinary bonds. The target distribution functions were obtained for the various bonds, angles, and dihedrals from the atomistic trajectory. In a couple of iterative steps, the CG parameters were adjusted to obtain the best match between the pseudo-CG and real-CG distributions. A full description of the CG topology and a comparison with atomistic data can be found in Figs. S1, S2, and Table S1. The SMA model is available at http://cgmartini.nl.

### Simulation details

We used the Martini 2.2P force field to model the interactions between lipid membranes and SMA copolymers (54, 57, 58). The primary setup consists of a bilayer composed of 1352 didecanoylphosphatidylcholine (DDPC) lipids built using the INSANE script (59) and 1, 10, or 20 SMA copolymers regularly arranged at a distance of 2.0 nm away from the lipid surface (Fig. 1 *b*). The solvent layer in those systems comprised between 24,527 and 38,526 water beads, wherein one bead represented four real water molecules. We used a concentration of 150 mM of sodium chloride, which is optimal for nanodisk formation according to previous experimental works (20, 27). All systems were neutralized by adding extra sodium ions. After minimization, all systems were first equilibrated at constant volume (NVT) and then at constant pressure (NPT, with semi-isotropic coupling) at a temperature of 310 K, using a Berendsen barostat and a V-rescale thermostat (60, 61). After equilibration, we changed the barostat to the Parrinello-Rahman method (62) while the standard Martini water model was still used, which proved to be more efficient at initiating the insertion of the polymers to the membrane surface (within a few hundred nanoseconds). We also applied a flat-bottomed potential on the copolymers to keep them close to the membrane solvent interface. The harmonic distance restraints keep the copolymers within a distance of 3.0 nm around the membrane surface, and the potential was released once the copolymers attached to the membrane. At this point, the standard water model was replaced by the polarizable Martini water model (63) to mimic the electrostatic interactions more realistically.

For each polymer concentration, we performed between two and five replicas starting from random initial velocities. Most simulations reached up to 3 *μ*s. To assess the thermodynamic stability of the nanodisks, self-assembly simulations were performed, starting from a random mixture of all the components, using 4, 8, or 16 SMA polymers with 600 lipids (corresponding to 150:1, 75:1, and 75:2 lipid ratios) and excess water. A polarizable water model was used, and two replicas were performed for each polymer/lipid ratio. All simulations were run using Groningen Machine for Chemical Simulations version 5.0.7 (64). The total simulation time covered over 60 *μ*s. To test the effect of polymer charge and lipid tail type, additional simulations were performed using 50% instead of fully charged polymers and longer-tail DMPC (dimyristoyl-PC), DPPC (dipalmitoyl-PC), or polyunsaturated dilinoleoyl-PC lipids. An overview of all simulations is provided in Table S2. Details of the atomistic simulations performed to calibrate the CG interactions can be found in the Supporting Materials and Methods.

## Results and Discussion

### SMA copolymers spontaneously inserted into the lipid bilayer

**Figure 2:**
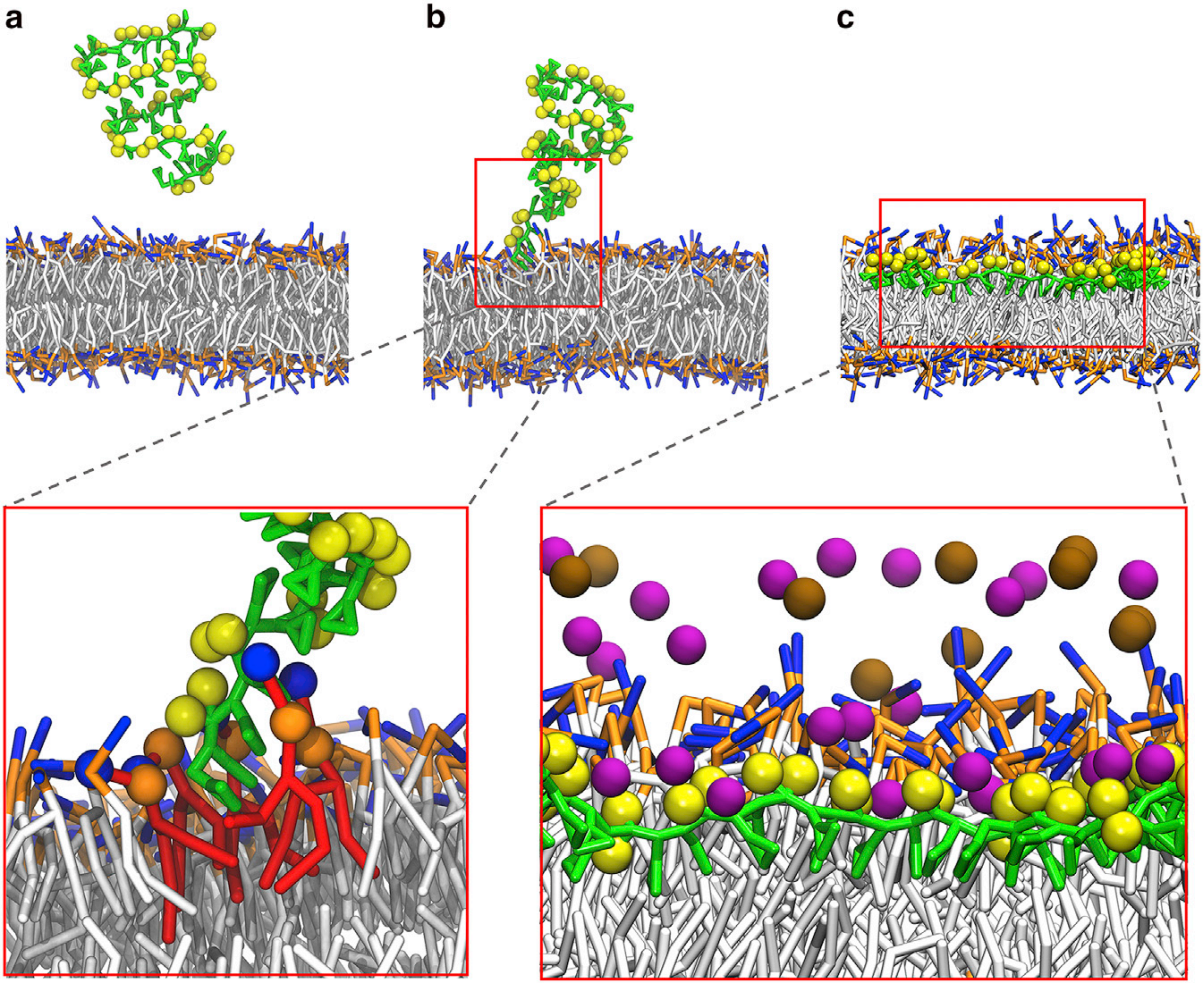
Snapshots of the binding process of an SMA copolymer to the surface of the membrane. (*a*) At the beginning (*t* = 0 ns), the polymer is in solution, taking a disordered conformation. (*b*) After 20 ns, the polymer adheres on the surface of the membrane through the hydrophobic terminal inserted between the lipid acyl tails. (*c*) At *t* = 400 ns, the polymer is fully absorbed to the lipid bilayer with sodium ions mediating the electrostatic interactions between the SMA carboxyl and the lipid headgroups. Styrene groups are shown in green, and carboxyl groups are shown in yellow. Lipids are shown with gray tails, orange phosphate, and blue choline groups. Lipids around the hydrophobic termini are highlighted in red in the left insert, with blue and orange beads representing the choline and phosphate groups, respectively. Purple beads represent Na^+^ ions, and brown beads represent Cl^−^ ions in the right insert. Some lipids in front of the polymer as well as water molecules were removed for clarity. Snapshots were obtained for a system with one copolymer. To see this figure in color, go online.

The starting setup of our simulations consisted of a bilayer composed of 1352 DDPC lipids. The short tail DDPC should facilitate membrane disruption on the accessible timescale of our simulations. We placed 1, 10, or 20 SMA copolymers in the aqueous phase in the vicinity of one of the membrane leaflets (Fig. 1 *b*). The initial asymmetric placement of the copolymers represents the experimental situation in which polymers are added in the solution surrounding a liposome or cell. Each SMA polymer consisted of 23 monomeric units and was fully deprotonated with two negative charges per monomer. The highly charged state mimics conditions of high pH, guaranteeing a highly soluble state of the polymers (27, 65), although the experimentally highest efficiency is obtained at somewhat lower pH (27). We performed multiple runs for each condition to increase the statistics (Table S2). In all cases, the SMA copolymers quickly adopted a disordered conformation in solution (Fig. 2 *a*), in agreement with potentiometric studies and with all-atom simulations (Fig. S3) (66). In the case of the simulation system with 10 or 20 SMA copolymers, the copolymers could also self-aggregate through their hydrophobic cores in solution, as shown experimentally (27).

The membrane affinity of the SMA copolymers, however, is high. We observed the spontaneous insertion of SMA copolymers already in the early phases (10–500 ns) of most of our simulations. Molecular insertion always started with the styrene moieties of the SMA polymeric termini (Fig. 2 *b*; Video S1). Hydrophobic interactions between the styrene moieties of the polymers and the lipid acyl chains seem to drive this behavior. The termini appeared to be strongly bound to the lipids because detachments were not observed after insertion. Once this is achieved, the rest of the copolymer slowly followed. This increases the interaction between the copolymer and the water-lipid interface. The inserted copolymers were located under the phosphate headgroups with the styrene moieties fitting between the acyl chains and the carboxyl groups pointing to the solution (Fig. 2 *c*). In the adsorbed state, the polymers became stretched. The analysis of the radius of gyration confirmed this change in structure upon membrane binding (Fig. S4). Counterions seem to play also an important role in stabilizing the polymer-lipid interactions (Fig. 2 *c*, *inset*). Analysis of the density profiles along the membrane normal revealed an asymmetric distribution of sodium ions around the membrane (Fig. S5 *a*). Insertion of SMA copolymers dragged additional sodium ions into the lipid/water interface. This seems to help the copolymers to overcome the repulsion between the charged carboxyl groups and the lipid phosphate groups (Fig. S5, *b* and *c*).

Video S1. Video of the Initial Binding Process of a SMA Copolymer to the Surface of the Membrane

### SMA copolymers perturbed the bilayer, inducing pore formation

**Figure 3:**
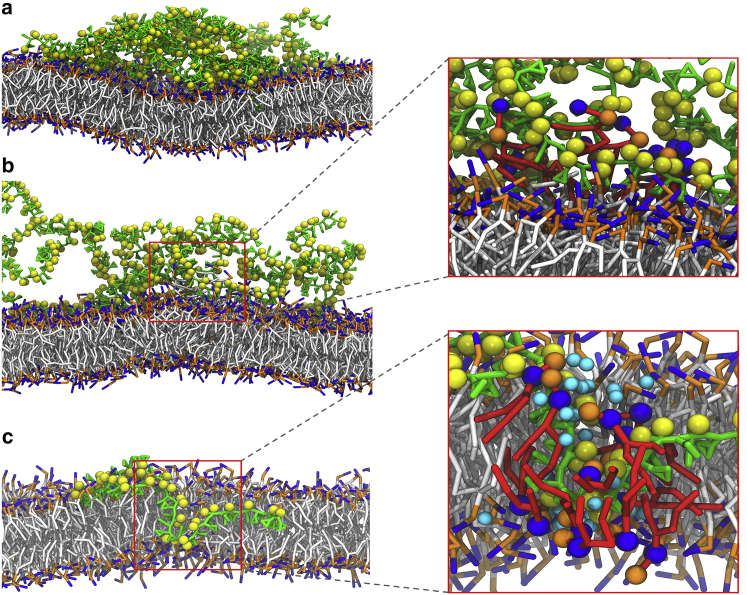
Membrane perturbations induced by SMA copolymers. (*a*) Membrane bending induced by the increased size of the hydrophobic core of the polymer cluster. (*b*) Lipids were pulled off from the membrane into the hydrophobic core of the polymer cluster. Extracted lipids are highlighted in red in the zoomed-in view. (*c*) Permeation of water molecules is shown close to an SMA copolymer (only one polymer chain shown). Colors are the same as in Figs. 1 and 2. Lipids around the water pore are highlighted in red in the zoomed-in view. Water molecules involved in the permeation through the membrane are depicted in cyan. In all cases, snapshots were obtained from independent simulations of a system containing 10 copolymers. To see this figure in color, go online.

The binding of SMA copolymers induced cooperative activities that included membrane bending, lipid extraction, lipid tilting, and water infiltration. In particular, when multiple polymers aggregated, the insertion of the SMA copolymers produced significant local bending of the membrane around the insertion site (Fig. 3 *a*). The bending originated from the increased size of the hydrophobic core in the leaflet to which they absorbed, causing stress and distorting the planarity of the lipid bilayer. In some simulations, the aggregate pulled lipids out of the membrane, ending up in the hydrophobic core of the polymers in solution (Fig. 3 *b*). This, however, might be facilitated by the short tail length of the lipids used and become more difficult with typical phospholipids. In most of our simulations, penetration of the copolymers caused infiltration of water molecules between the lipids’ tails. This made the lipids close to the copolymers tilt and shield their tails from the carboxyl groups of the copolymers and the water molecules. Some lipids even toppled over, lying horizontal to the membrane surface (Fig. 3 *c*). The disorder in the lipid bilayer allowed other water molecules on the other side to cross the lipid bilayer, forming transmembrane pores. At the same time, the polymers bridged to the other side, spanning across the membrane (Fig. 3 *c*). Together, the lipid flip-flopping and polymer translocation relieved the stress imbalance induced by the asymmetric adsorption of the SMA copolymers. Again, the timescale of this process is likely dependent on the length of the lipid tail and artificially enhanced in our simulations.

### SMA stabilized the pore rim, resulting in pore growth and membrane disruption

**Figure 4:**
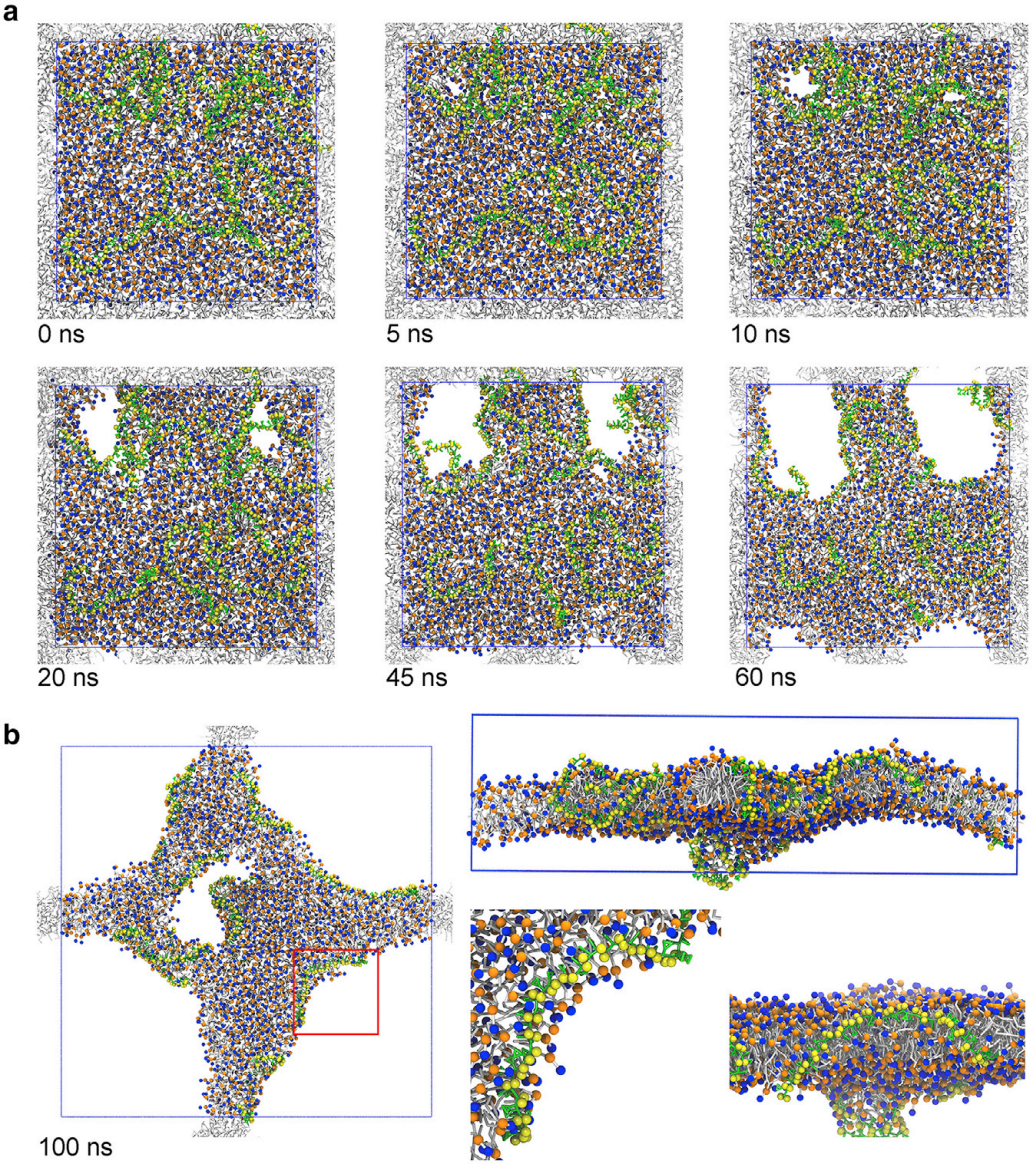
Pore formation upon SMA copolymer binding to the lipid bilayer. (*a*) A top view of the process shows first the SMA binding (*t* = 0 ns) and the initial water permeation (*t* = 5 ns), followed by the pore formation (*t* = 10 ns) and growth of those pores in the membrane (*t* = 20–60 ns) for a system containing 10 copolymers. The simulation times are shown in each figure, with the time of the first snapshot reset to zero. (*b*) The final snapshot of the simulation (*t* = 100 ns) is shown from both top and side views. The pore edge is shown in more detail at the bottom right (*top view* and *side view*). SMA copolymers are shown in green and yellow. Lipids are depicted with gray tails and orange/blue spheres for phosphate/choline groups. Water molecules are removed for clearness. Periodic boundary boxes are shown as blue grid lines. Lipids in other periodic boxes are shown in gray. To see this figure in color, go online.

Even though the initial stress imbalance largely dissipated, water permeation increased after the initial transmembrane pores formed. The amphipathic nature of SMA copolymers would likely favor the interaction with the water molecules inside the pore and the lipid tails. This further stabilized the pore’s rim. The hydrophobic styrene groups intercalated perpendicularly to the lipid acyl chains, which agrees with the polarized attenuated total reflection Fourier-transform infrared spectroscopy measurements (9). The SMA carboxyl groups and the nearby lipid headgroups faced toward the water pore. This forced the lipids around the pore to tilt, forming a toroidal pore. At the beginning, the pores showed a roughly cylindrical shape and became more irregular as the pores expanded (Fig. 4 *a*). At the end of the simulations with high concentration of SMA copolymers, we observed big pores forming (with diameters of 5–10 nm) and the original bilayer largely destroyed (see Fig. 4 *b*). At this point, the systems seemed to reach a metastable state, preventing the complete formation of nanodisks. It is possible that the periodic boundary conditions used in the simulation artificially stabilized the connectivity in the plane of the membrane, resulting in a kinetic trap. The formation of the full pore upon SMA copolymer binding is shown in Video S2. We also quantified the kinetics of pore expansion by measuring the sizes of several pores over time (Fig. S6 *c*).

Video S2. Video of Pore Formation and Membrane Disruption by Multiple Absorbed SMA Copolymers

### Complete SMALP nanodisks formed by self-assembly

**Figure 5:**
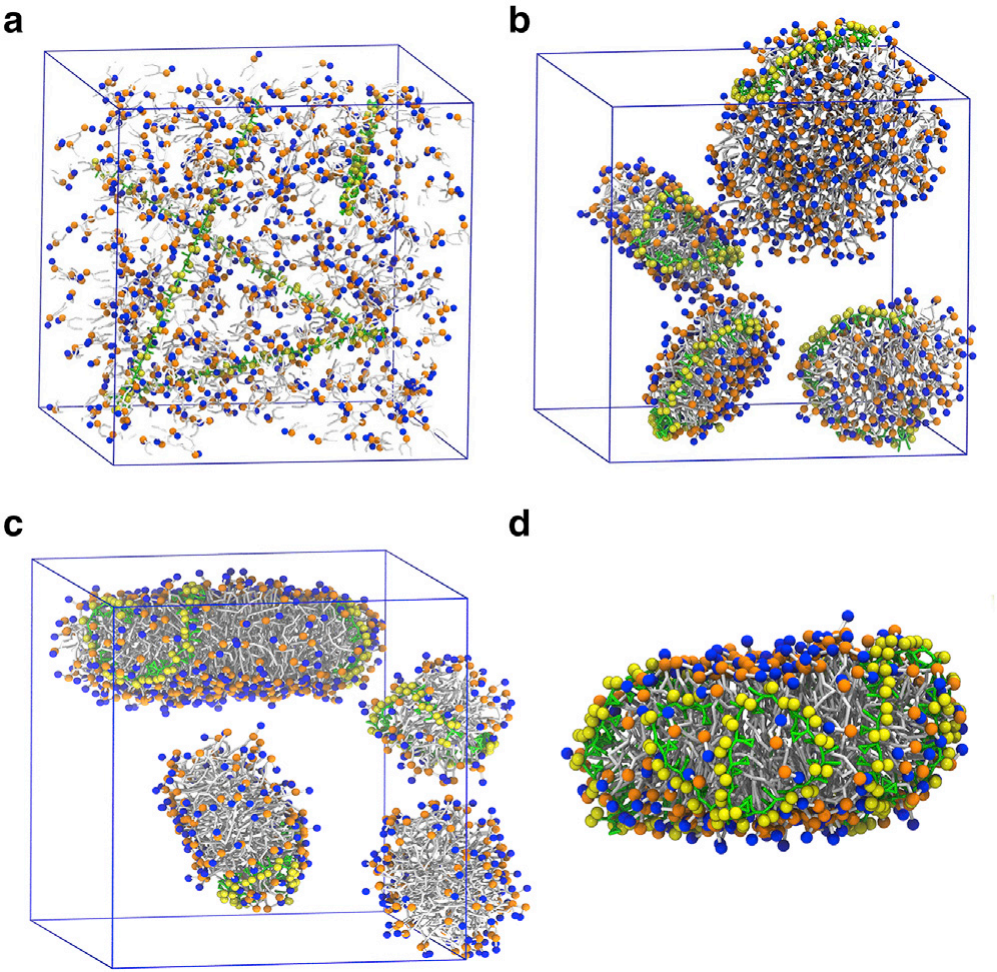
Formation of SMALPs in the self-assembly experiment. (*a*) The initial random distribution of DDPC lipids and SMA copolymers is shown at a 150:1 SMA/lipid ratio. (*b*) The resulting SMALP nanodisks are shown. (*c*) SMALP nanodisk and micelles are formed in self-assembly simulation using DPPC lipids. (*d*) A snapshot is shown of a single nanodisk surrounded with multiple copolymers, formed in SMA and DPPC self-assembly simulation at a 75:2 SMA/lipid ratio. The same coloring is used as in the other figures. To see this figure in color, go online.

To test the capability of SMA copolymers to form stable SMALPs and to avoid metastable states in preformed membrane bilayers, we also performed self-assembly experiments. We used a mixture of SMA copolymers and DDPC lipids (either 4, 8, or 16 SMA copolymer molecules per 600 lipid molecules), which corresponds to 150:1, 75:1, or 75:2 lipid/polymer ratios. Without copolymers, lipids form stable bilayers in self-assembly simulations (57). However, when we added SMA copolymers to DDPC lipids, stable SMALPs formed with the SMA copolymer bound to the edge of the lipid bilayer disk (Fig. 5). Often a few micelles initially also remained present. As the simulations progressed, however, these micelles merged with other nanodisks through the exposed polymer-depleted sides. The formed nanodisks were always stable. The embedding of the SMA copolymers during the self-assembly simulations was similar to what we observed during the membrane-disruption simulations. SMA copolymers stabilized the pore rim with the styrene and the carboxyl groups in opposite directions (Fig. 4 *b*).

In the self-assembly simulations with a 150:1 lipid/polymer ratio, the SMA copolymers formed four nanodisks, with one polymer chain per nanodisk. The diameters of the nanodisks ranged from 7 to 9 nm. This agrees with the overall structural measurements of free SMALPs in solution. Small-angle neutron scattering measurements have shown that the inner radius of SMA nanodisks is around 3.8 ± 0.2 nm (10). The nanodisks comprised one polymer in their annulus, in line with experimental data showing nanodisks surrounded by a one polymer thick belt (9). Despite the use of a different lipid composition in those experiments, earlier experiments have shown that the particle shape and the average diameter of the nanodisks are independent of the acyl-chain length (20).

To test whether SMA copolymers can form nanodisks with long-tail lipids, we performed additional self-assembly simulations, replacing DDPC with either DMPC (myristoyl chains, 14 carbons) or DPPC (palmitoyl chains, 16 carbons) lipids. In both cases, stable nanodisks formed with similar sizes and lipid/copolymer ratios compared to DDPC (Fig. S7). With DPPC, however, we often observed two SMA polymer chains per nanodisk, which can be explained by the larger size of the hydrophobic core (Fig. 5 *c*). To further investigate the polymer concentration effect on the nanodisks formed, we also performed self-assembly simulations at higher (75:1 and 75:2 lipid/polymer ratio) polymer concentrations. Again, nanodisks formed (Fig. S7), but several SMA copolymers surrounded the lipid disk, in agreement with the general idea that multiple polymer chains are required to completely surround a nanodisk (67). Fig. 5 *d* shows an example of such a nanodisk.

### Limitations of our model and further controls

Simulations of membrane solubilization at an all-atom level of resolution are computationally too expensive and cannot currently be performed. The use of a CG model allows such computations but implies that some detail is lost. An extensive discussion of the assumptions and limitations underlying the Martini model can be found in (36). Here, we briefly discuss the main limitations that could influence our results. One such limitation is the directionality of hydrogen bonds, which is missing in the Martini model; hydrogen bonds are represented isotropically only. Previous work, however, indicates that this is not an important limitation in capturing the essence of membrane-polymer interactions (37, 38, 39, 40, 41, 42, 43, 44, 45, 46, 47, 48, 49, 50, 51, 52). Another limitation, in particular when compared to experimental settings, is the small system sizes considered in this study combined with periodic boundary conditions. The latter may cause an artificial stabilization of the lamellar phase, which makes it more difficult for the polymers to break the membrane into nanodisks. Together with the limited timescales that can be reached by our simulations (microsecond range), this prompted us to select the short-tail DDPC lipids to speed up the process. Energy barriers for pore formation in longer-tail lipids are large, estimated to be around 45 and 78 kJ/mol for DMPC and DPPC, respectively, according to previous all-atom simulations (68). Pore formation is even harder to observe in Martini CG simulations (69). Additional simulations using longer-tail lipids, DMPC or DPPC, and the polyunsaturated dilinoleoyl-PC lipids revealed that the initial adsorption process of the polymers was similar to what we observed for DDPC lipids (Fig. S8), but pores did not form. Therefore, pore formation probably requires longer timescales. However, we expect the mechanism of SMALP formation to be generic. Reassuringly, we observed similar nanodisks forming upon self-assembly, comparing short-tail lipids to more common longer-tail lipids, as shown in Figs. 5 and S7. This data suggests that rupture of intact membranes of longer-tail lipids eventually will also happen with our models but perhaps requires the use of smart sampling techniques to observe the process of pore formation.

Another important difference regarding typical experimental settings is the optimal polymer charge density and lipid/polymer ratio. To probe the effect of polymer charge, we performed additional simulations using 50% charged SMA copolymers (i.e., every second maleic acid unit was considered protonated). Experimental data suggest that dissociation of ∼50% is more appropriate (27). Our simulations showed that changing the charged state of the polymer did not lead to qualitative differences for those conditions tested (see Fig. S6). One difference, though, was that the pores expanded at different rates, with the fully charged model expanding faster than the half-protonated one (Fig. S6, *c* and *d*), probably due to the larger charge density inside the pore in the case of the fully charged model. Concerning the lipid/polymer ratio, the experiments show that nanodisk formation is more efficient at lipid/polymer weight ratios of between 1:1 and 1:3, depending on the type of copolymers (16). However, control simulations with higher polymer concentrations (up to ∼3:1 lipid/polymer weight ratio) led to extensive clustering of the polymers in the aqueous phase (Fig. S9). This clustering behavior severely hampered the adsorption and insertion efficiency of the polymers into the membrane. On an experimental timescale, this is no problem, but on our simulation timescale, it is. Fortunately, using the self-assembly setup, it was possible to explore higher polymer concentrations, revealing nanodisk formation at a 75:2 lipid/polymer molar ratio (∼3:2 weight ratio), with multiple polymers stabilizing the rim (Fig. 5 *d*).

## Conclusions

We investigated the molecular mechanism of the early stages of SMA nanodisk formation using CG MD simulations. Despite the limitations associated with our model, we expect our findings to be generic. More detailed all-atom models should validate our results. Based on our simulations, we propose the following mechanism for SMA-induced nanodisk formation:

1. SMA copolymers bind to the membrane surface through the styrene moieties of the termini. The hydrophobic interactions drive the initial insertion with the core of the lipid bilayer.
2. Full insertion of the SMA copolymers’ hydrophobic side chains follows, causing local membrane undulation.
3. Translocation of the SMA copolymers relieves the induced stress, together with water molecules and accommodated by lipid flip-flop. Small transmembrane pores form.
4. Growth of the transmembrane pores occurs. The SMA copolymers stabilize the rim by orienting the carboxyl moieties to the water pore, and the benzene groups intercalated in between the lipid tails. This likely disrupts the membrane and favors nanodisk formation. Because of periodic boundary effects, we could not observe the last phase, but self-assembly simulations show that SMALP nanodisks are the thermodynamically favorable state of the system.

Our findings show the solubilization ability of SMA copolymers and the details of the process at the molecular level. Our simulation protocol paves the way for further studies of SMA nanodisks, exploring different conditions (pH, polymer composition, and multicomponent lipid membranes) and will help the design of optimized copolymers for nanodisk formation and drug-delivery systems. Simulations of nanodisk formation with membrane-embedded proteins are underway. They will contribute to understanding the influence of SMA copolymers on the structural and dynamic properties of proteins and their annular lipid shells in SMA nanodisks.

## Author Contributions

M.X., I.F., and S.J.M. designed the research. M.X. and L.C. performed the research. M.X. and I.F. analyzed the data. M.X., I.F., W.G., and S.J.M. wrote the article.

## Supporting Material

Document S1. Supporting Materials and Methods, Figs. S1–S9, and Tables S1 and S2

Document S2. Article plus Supporting Material
