# Supplementary material for: Molecular Mechanism of Lipid Nanodisk Formation by Styrene-Maleic Acid Copolymers
Source: Biophys J. 2018 Jun 20;115(3):494–502. doi: 10.1016/j.bpj.2018.06.018 (PMC6084417; doi:10.1016/j.bpj.2018.06.018)
Supplement: Document S1. Supporting Materials and Methods, Figs. S1–S9, and Tables S1 and S2 [file mmc1.pdf]

Biophysical Journal, Volume *115*

Supplemental Information

Molecular Mechanism of Lipid Nanodisk Formation by Styrene-Maleic Acid Copolymers

Minmin Xue, Lisheng Cheng, Ignacio Faustino, Wanlin Guo, and Siewert J. Marrink

Supplementary Information

Molecular Mechanism of Lipid Nanodisc Formation by Styrene Maleic Acid Copolymers

Minmin Xue[1,2,3], Lisheng Cheng[4], Ignacio Faustino[2,3], Wanlin Guo[1], and Siewert J. Marrink*[2,3].

[1]State Key Laboratory of Mechanics and Control of Mechanical Structures and Key Laboratory for Intelligent Nano Materials and Devices of the Ministry of Education, and Institute of Nanoscience, Nanjing University of Aeronautics and Astronautics, Nanjing 210016, People's Republic of China.

[2]Groningen Biomolecular Science and Biotechnology Institute, University of Groningen, Nijenborgh 7, 9747 AG, Groningen, The Netherlands.

[3]Zernike Institute for Advanced Materials, University of Groningen, Nijenborgh 4, 9747 AG, Groningen, The Netherlands.

[4]College of Mechanical and Electrical Engineering, Beijing University of Chemical Technology, Beijing 100029, People's Republic of China

SUPPLEMENTARY METHODS

Parametrization and validation of the Martini SMA model

Quantum mechanics calculations: The chirality sequence in short SMA polymers has an effect on the conformational properties of the linear polymer[1]. Based on preliminary atomistic simulations of short SMA chains with different chirality, we noticed that the bonded distributions showed better convergence using a tetramer. To predict the most stable stereo isomeric tetramers, we used quantum mechanics calculations for all possible stereoisomers (2^4) (see Fig. S1). We used a short chain of styrene and maleic anhydride copolymer since this is the precursor before hydrolysis during the industrial production of the SMA-2000 copolymer. We used the semi-empirical PM6 method because it accounts for accurate intramolecular interactions and has the advantage of being less time-consuming than other DFT methods. The conformational search of each tetramer was performed in the gas phase at the PM6 level of theory. Eventually, the five most stable chirality sequences were used for the parametrization of the coarse-grained (CG) SMA model.

Mapping: The mapping of the CG beads was chosen based on the mapping of previous Martini models (see Fig. 1). For the styrene group, a three-beads mapping scheme was used similar to the ring-based side chains in the aromatic phenylalanine and tyrosine amino acids and the styrene group in the Martini polystyrene model. For the maleic acid group, a one-bead representation was used to represent the carboxylic group, carrying a full negative charge each. The full SMA model is available at http://cgmartini.nl.

Bonded and non-bonded parameters of the Martini SMA model: Biphasic simulations were used for the parametrization of the Martini SMA model. Based on the semi-empirical quantum mechanics calculations, we built five different SMA copolymers based on the most stable SMA tetramers (see Table S1). In these simulations, one SMA copolymer was placed at the interface of water and dodecane. The system size was set to 4.4×4.4×6.9 nm^3 and a similar number of water and dodecane molecules were included. Sodium ions were added to neutralize the negative charge of the SMA copolymer. Bonds, angles and proper and improper dihedral angles of the CG molecules were optimized by comparison with atomistic simulations using the OPLS-AA force field[2]. The details of the CG simulations can be found in the *Methods* section of the main manuscript. The target distribution functions were obtained after a few iterative steps and were agree remarkably well with the atomistic distributions (Fig. S2a). We tested the CG models by calculating the radii of gyration depending on polymer chain length, and by comparing the results obtained from CG and AA simulations (Fig. S2b). A very good agreement between the CG and AA models was obtained.

SMA copolymers in solutions: To further validate the CG model, we considered the behavior of the full SMA copolymer chain in solutions. Here we compared our CG model with an atomistic model using the CHARMM36 force parameters set[3]. The system consists of a single SMA chain of 23 monomeric units in excess aqueous solvent. Constant temperature was maintained at 310 K and constant pressure was maintained at 1 atm. Full electrostatic forces were evaluated using the particle-mesh Ewald method with a cutoff of 1.4 nm[4]. Short non-bonded terms were evaluated every step using a cutoff of 1.4 nm for van der Waals interactions. The last 200 ns of the simulation were used for data analysis, with the first 50 ns considered as equilibrium. Previous potentiometric titration studies of SMA copolymers showed that, at neutral and high pH, the electrostatic repulsions between the carboxylic groups dominate the hydrophobic interactions between styrene groups within the polymers, resulting in a disordered conformation that dissolves relatively easily in aqueous solution[5, 6]. Both our all-atom and CG simulations showed that the fully charged SMA copolymer adopts a mostly disordered conformation in agreement with the potentiometric studies (Fig. S3a,b). The conformations adopted by the polymers range from partly collapsed to full extended. Visual inspection indicates also the presence of loops forming within one copolymer, providing some shielding of the styrene moieties (see Fig. 2A in the main manuscript). Both atomistic and CG MD simulations of the SMA copolymer showed similar flexibility properties according to the end-to-end distances and radius of gyration (Fig. S3c and S3d).

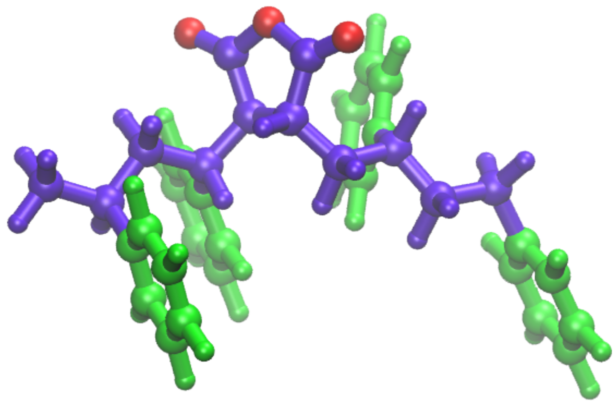

Figure S1 | The structure of a short chain of styrene-maleic anhydride copolymer used to calculate the configurational energy with quantum mechanics.

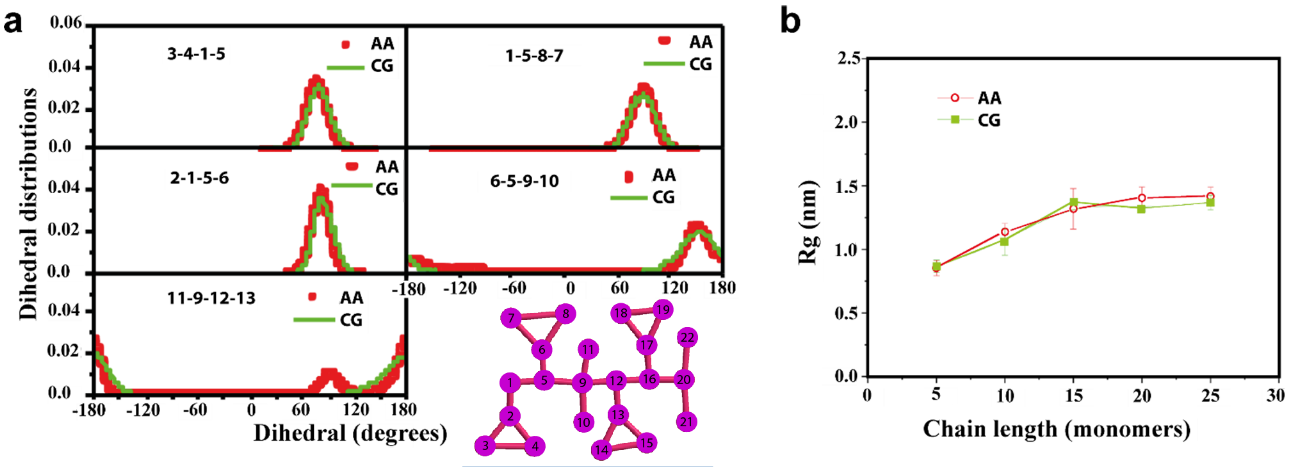

Figure S2 | Analysis of the Martini SMA model and comparison with OPLS-AA atomistic simulations. (**a**) Distributions of dihedral angles obtained from atomistic simulations and optimized coarse grain parameters. The numbers correspond to the beads in CG topology. (**b**) Radius of gyration depending on the polymer chain length. The copolymer is composed of the five monomer types in a random order.

Chirality sequence	Configurational energy (kJ/mol)
RRRR	-0.53
RRRL*	-10.85
RRLR*	-14.31
RRLL	3.37
RLRR	21.44
RLRL	8.98
RLLR	0.79
RLLL	21.44
LRRR	-1.05
LRRL*	-13.34
LRLR*	-17.94
LRLL*	-11.55
LLRR	10.0
LLRL	2.64
LLLR	-1.84
LLLL	2.68

Supplementary Table S1 | Energies of the sixteen chirality sequences of optimized geometries of *styrene-styrene-maleic acid-maleic acid* tetramers. Configurational energies were calculated using the PM6 semi-empirical method. (*) Configurations corresponding to the lowest configurational energies among the 16 combinations. L and R denote the left and right chiralities of the SMA units, respectively.

Type of simulation	Lipid type	Copolymer type (percentage charged)	Number of SMA copolymers/lipids	Number of replicas	Simulation length (us)	Number of pores (bilayer) / nanodiscs (self-assembly) formed
Bilayer	DDPC	100%	1 / 1352	2	2.4	0 / 0
			10 / 1352	5	3.0	2 / 3 / 3 / 2 / 3
			10 / 1352	3	0.5	0 / 1 / 0
			20 / 1352	2	0.4	1 / 0
			40 / 1352	2	0.2	0 / 0
		50%	10 / 1352	2	1.0	3 / 4
	DMPC	100%	1 / 1352	2	1.0	0 / 0
			10 / 1352	2	2.0	0 / 0
			20 / 1352	1	1.0	0
		50%	10 / 1352	1	1.0	0
	DPPC	100%	1 / 1352	2	1.0	0 / 0
			10 / 1352	2	2.0	0 / 0
		50%	10 / 1352	1	1.0	0
	DIPC	100%	10 / 1352	2	3.0	0 / 0
			20 / 1352	2	2.0	0 / 0
		50%	10 / 1352	1	1.0	0
Self-assembly	DDPC	100%	4 / 600	2	2.0	4 / 4
			8 / 600	2	0.9	5 / 7
			16 / 600	2	1.0	11 / 12
		50%	16 / 600	1	1.0	9
	DMPC	100%	4 / 600	2	2.0	4 / 3
			8 / 600	2	1.0	1 / 3
			16 / 600	2	1.0	7 / 8
		50%	16 / 600	1	1.0	8
	DPPC	100%	4 / 600	2	2.0	4 / 3
			8 / 600	2	1.0	4 / 5
			16 / 600	2	1.0	6 / 7
		50%	16 / 600	1	1.0	7

Table S2. Details and outcomes for bilayer and self-assembly simulations used in this work. Only bilayer simulations with regularly placed SMA copolymers are listed here.

SUPPLEMENTARY RESULTS

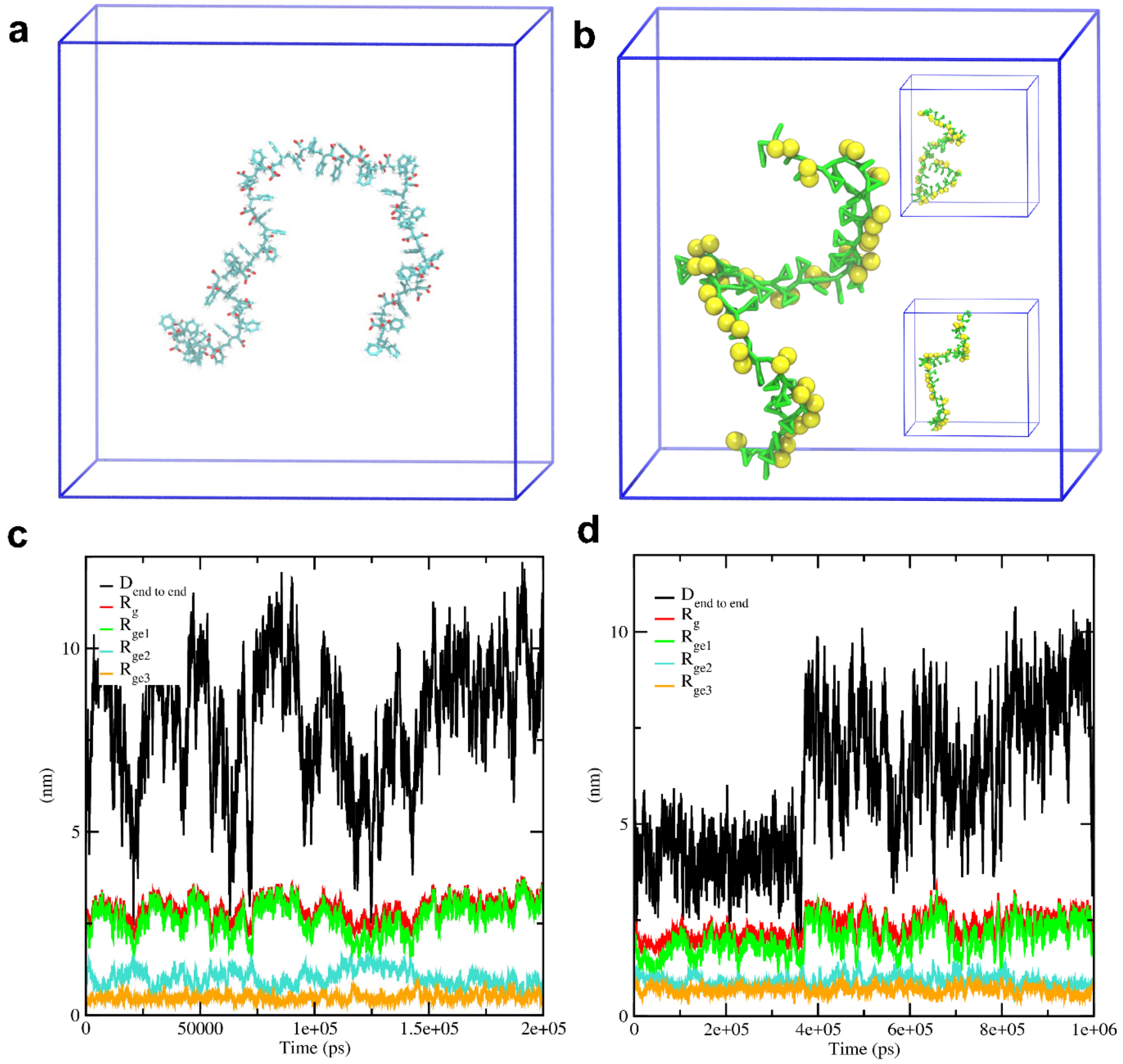

Figure S3 | Comparison of the polymer flexibility properties obtained from atomistic (left) and coarse grain (right) simulations of SMA copolymers in aqueous solvent. In the upper part, a schematic view of SMA copolymers in (**a**) all-atom and (**b**) coarse-grained representations is shown. The bottom row shows the time evolution of the end-to-end distances and radii of gyration in (**c**) atomistic and (**d**) coarse-grained MD simulations. The jump in the end-to-end distance in (**d**) is due to a conformational change from a partially collapsed to a fully stretched out conformation, as is shown in the insets in (**b**).

Effect of SMA copolymer adsorption on membrane properties and polymer end-to-end distance

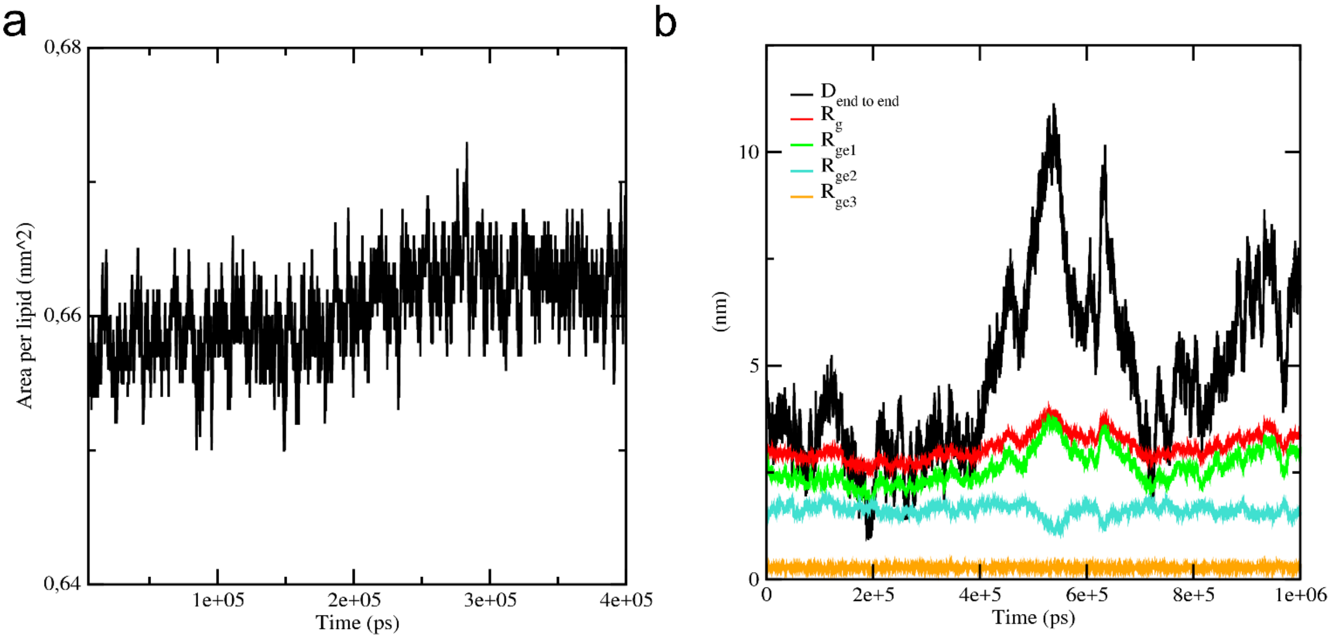

Figure S4 | Analysis of some properties of the membrane and the SMA copolymer during the attachment process. (**a**) The increase in the area per lipid is due to the binding and lying down of the SMA copolymer on the surface of membrane. (**b**) Change in the end-to-end distance and radius of gyration of the SMA copolymer. The results correspond to one of the simulations with 1 SMA copolymer. Similar results were obtained in other simulations at high SMA concentration.

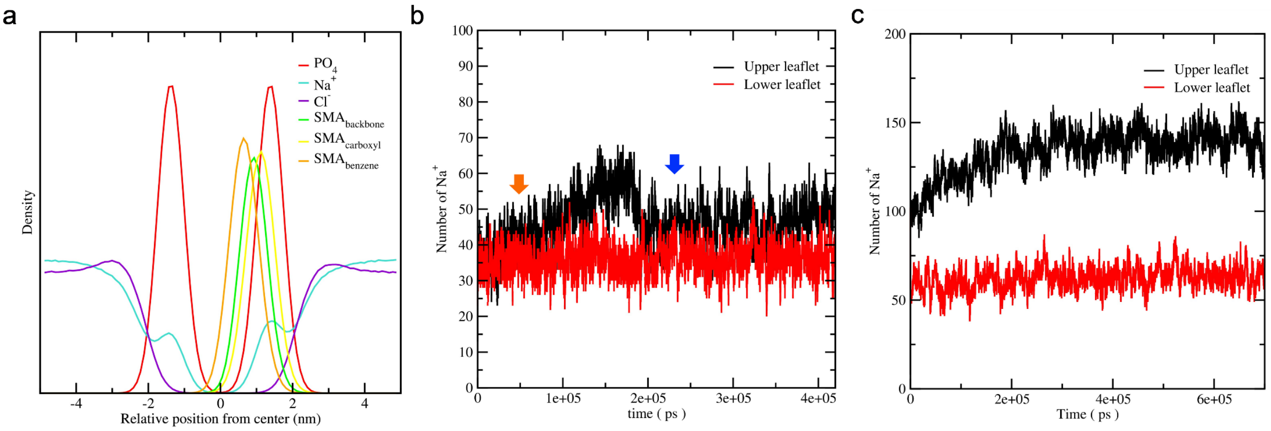

Figure S5 | Density profiles across the z-axis and sodium ion distribution in the lipid/water interface. (**a**) Density profiles across the z-axis of the membrane after the SMA copolymer is attached in CG simulations. The benzene rings insert deeper into the hydrophobic core while the carboxyl groups point towards the solvent. The results correspond to one of the simulations with 1 SMA copolymer. Similar results were obtained in the other simulations at high SMA concentration. (**b**) Time evolution of number of Na^+ ions within 7 Å of the phosphate head group on both sides of the DTPC membrane in the simulation with 1 SMA

copolymer on top. The orange and blue arrows indicate the time the SMA copolymer starts to insert into the membrane and becomes fully stretched out on the surface, respectively. (c) Time evolution of number of Na^+ ions upon adsorption on both sides of the DTPC membrane in system with 10 SMA copolymers on top of the upper leaflet.

Simulations of half charged SMA copolymers

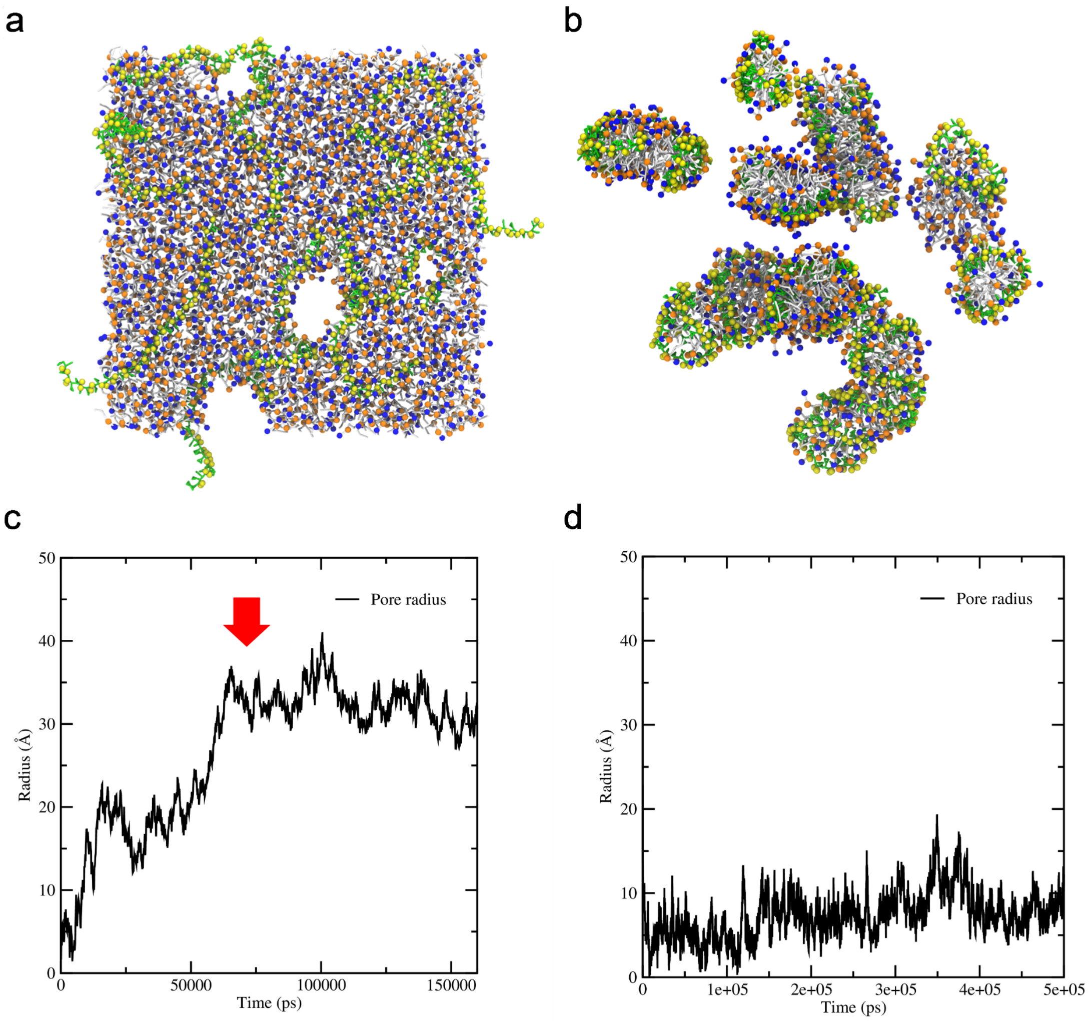

Figure S6 | Simulation results of half charged copolymers. (**a**) Snapshot of the simulation using 10 SMA copolymers and DDPC bilayer at 500 ns. (**b**) Self-assembly simulation of DDPC SMALPs using 2:75 polymer-to-lipid ratio. (**c**) Radius for one of the pores in the disrupted DDPC bilayer from fully charged model. The pore stops growing until trapped in a metastable state (red arrow). (**d**) Radius for one of the pores in the disrupted DDPC bilayer from half charged model.

Self-assembly process for nanodiscs of DDPC, DMPC and DPPC lipids

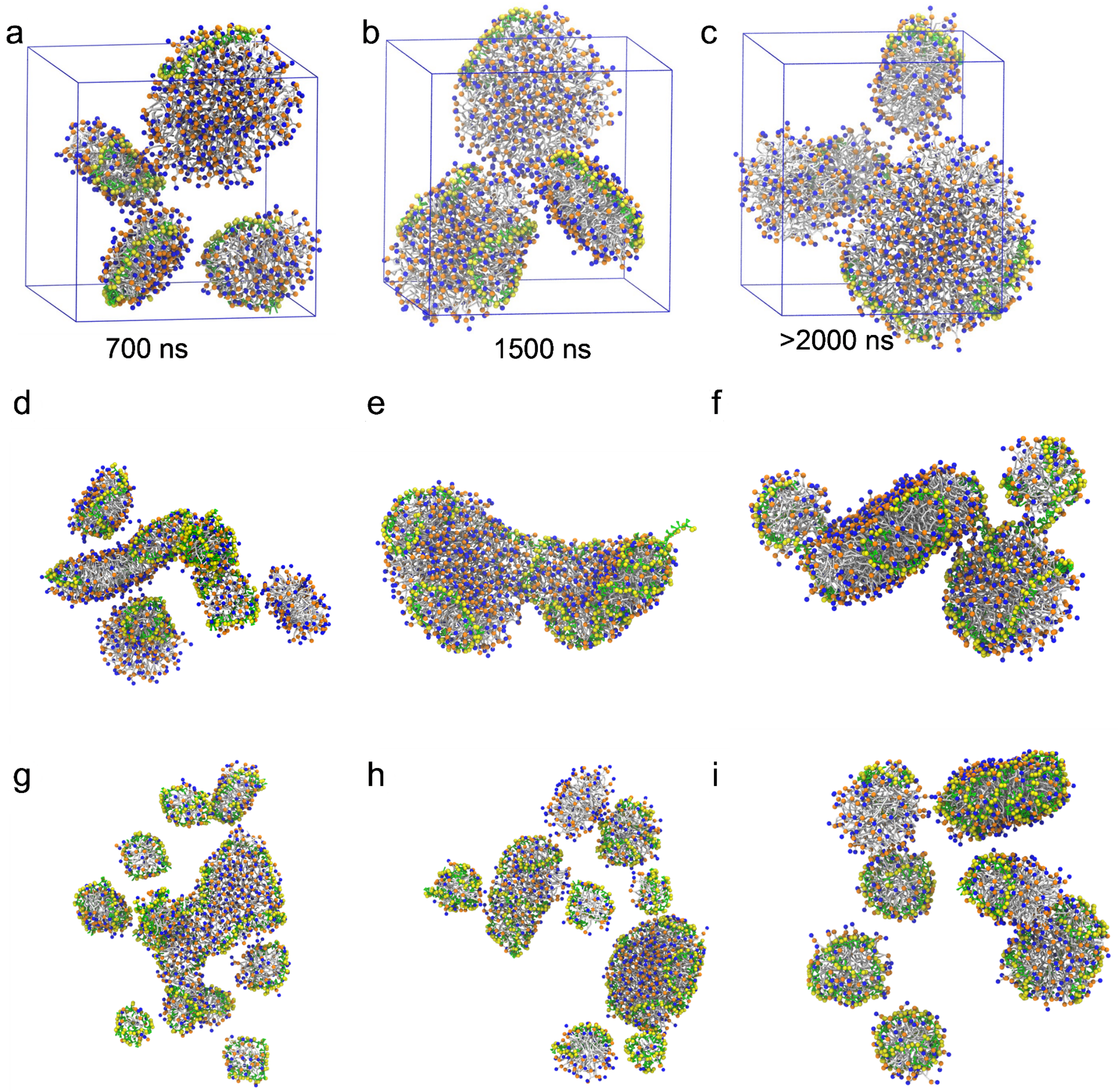

Figure S7 | Formation of SMALPs during self-assembly experiments with DDPC, DMPC and DPPC lipids using CG simulations. (**a**) DDPC SMALPs, (**b**) DMPC SMALPs and (**c**) DPPC SMALPs assemblies after 2 microsecond simulation with polymer-to-lipid ratio of 1:150. PBC boxes are shown in blue grid lines. Simulation time required for nanodiscs self-assembly are shown for each system. (**d**) DDPC SMALPs, (**e**) DMPC SMALPS and (**f**) DPPC SMALPs assemblies after 1 microsecond simulation with polymer-to-lipid ratio of 1:75. (**g**) DDPC SMALPs, (**h**) DMPC SMALPs and (**i**) DPPC SMALPs assemblies after 1 microsecond simulation with polymer-to-lipid ratio of 2:75.

Adsorption of SMA copolymers on top of long tail lipid membrane surface

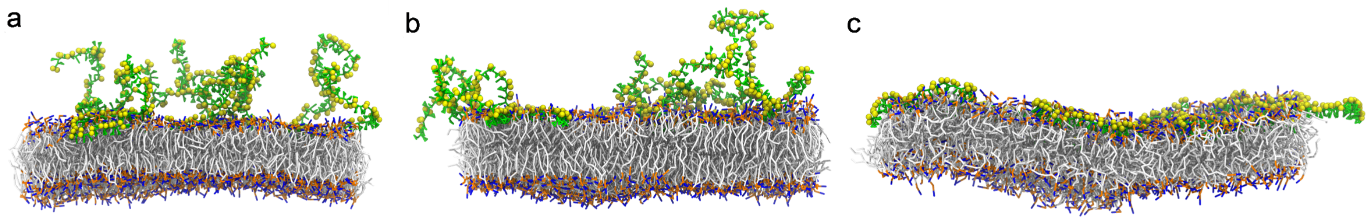

Figure S8 | Snapshots of adsorption of 10 SMA copolymers on DMPC, DPPC and DLiPC membrane surface after 2 us simulation. (**a**) Adsorption of 10 SMA on DMPC membrane. (**b**) Adsorption of 10 SMA on DPPC membrane. (**c**) Adsorption of 10 SMA on DLiPC membrane. Solvents are omitted for clarity. Some copolymers are fully adsorbed while some still have part of it in solution. Faster and complete adsorption of SMA copolymers are observed for DLiPC membrane, which indicates DLiPC could be a potential candidate for pore formation in nanodisc computational research.

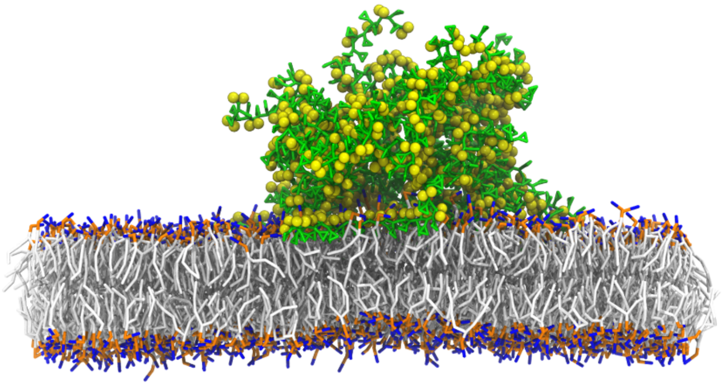

Figure S9 | Snapshots of adsorption of 20 SMA copolymer on DMPC membrane surface. Cluster of aggregated 20 SMA were formed and adsorbed on surface of DMPC membrane after 1 μs simulation. Solvents are omitted for clarity.

Supporting Citations

1. Malardier-Jugroot C, van de Ven TGM, Whitehead MA. Linear conformation of poly (styrene-*alt*-maleic anhydride) capable of self-assembly: a result of chain stiffening by internal hydrogen bonds. *The Journal of Physical Chemistry B* **109**, 7022-7032 (2005).

2. Jorgensen WL, Maxwell DS, Tirado-Rives J. Development and Testing of the OPLS All-Atom Force Field on Conformational Energetics and Properties of Organic Liquids. *Journal of the American Chemical Society* **118**, 11225-11236 (1996).

3. Vanommeslaeghe K*, et al.* CHARMM general force field: A force field for drug-like molecules compatible with the CHARMM all-atom additive biological force fields. *Journal of computational chemistry* **31**, 671-690 (2010).

4. Essmann U, Perera L, Berkowitz ML, Darden T, Lee H, Pedersen LG. A smooth particle mesh Ewald method. *The Journal of Chemical Physics* **103**, 8577-8593 (1995).

5. Ohno N, Nitta K, Makino S, Sugai S. Conformational transition of the copolymer of maleic acid and styrene in aqueous solution. *Journal of Polymer Science Part A-2: Polymer Physics* **11**, 413-425 (1973).

6. Tonge SR, Tighe BJ. Responsive hydrophobically associating polymers: a review of structure and properties. *Advanced Drug Delivery Reviews* **53**, 109-122 (2001).
