# Supplementary material for: Molecular Mechanism of Lipid Nanodisk Formation by Styrene-Maleic Acid Copolymers
Source: Biophys J. 2018 Jun 20;115(3):494–502. doi: 10.1016/j.bpj.2018.06.018 (PMC6084417; doi:10.1016/j.bpj.2018.06.018)
Supplement: Document S2. Article plus Supporting Material [file mmc4.pdf]

Biophysical Journal
Article

Biophysical Society

Molecular Mechanism of Lipid Nanodisk Formation by Styrene-Maleic Acid Copolymers

Minmin Xue,[1,2,3] Lisheng Cheng,[4] Ignacio Faustino,[2,3] Wanlin Guo,[1] and Siewert J. Marrink[2,3,*]
[1]State Key Laboratory of Mechanics and Control of Mechanical Structures, Key Laboratory for Intelligent Nano Materials and Devices of the Ministry of Education, Institute of Nanoscience, Nanjing University of Aeronautics and Astronautics, Nanjing, People's Republic of China; [2]Groningen Biomolecular Science and Biotechnology Institute, University of Groningen, Groningen, the Netherlands; [3]Zernike Institute for Advanced Materials, University of Groningen, Groningen, the Netherlands; and [4]College of Mechanical and Electrical Engineering, Beijing University of Chemical Technology, Beijing, People's Republic of China

ABSTRACT Experimental characterization of membrane proteins often requires solubilization. A recent approach is to use styrene-maleic acid (SMA) copolymers to isolate membrane proteins in nanometer-sized membrane disks, or so-called SMA lipid particles (SMALPs). The approach has the advantage of allowing direct extraction of proteins, keeping their native lipid environment. Despite the growing popularity of using SMALPs, the molecular mechanism behind the process remains poorly understood. Here, we unravel the molecular details of the nanodisk formation by using coarse-grained molecular dynamics simulations. We show how SMA copolymers bind to the lipid bilayer interface, driven by the hydrophobic effect. Due to the concerted action of multiple adsorbed copolymers, large membrane defects appear, including small, water-filled pores. The copolymers can stabilize the rim of these pores, leading to pore growth and membrane disruption. Although complete solubilization is not seen on the timescale of our simulations, self-assembly experiments show that small nanodisks are the thermodynamically preferred end state. Our findings shed light on the mechanism of SMALP formation and on their molecular structure. This can be an important step toward the design of optimized extraction tools for membrane protein research.

INTRODUCTION

Membrane proteins are of great importance to a variety of essential physiological functions in all organisms. Encoded by 30% of all genes, membrane proteins account for almost 70% of known drug targets in the cell. However, they only contribute less than 2% of the structures in the Protein Data Bank (1). These proteins are relatively less studied because of a lack of experimental approaches. One of the major challenges in membrane protein research is the isolation of these proteins without destroying their stability and activity. Extraction of membrane proteins from their lipid environments can lead to their inactivation or aggregation.

A widely used solution is to incorporate the protein into a model lipid membrane. In particular, lipid nanodisks have proven to be an efficient way to solubilize membrane proteins while keeping a natural environment (2–6). In the pioneering work of Sligar and co-workers, these small bilayer patches are surrounded and stabilized by a ring of α-helical peptides (also called membrane scaffold proteins (MSP)) (7,8). One disadvantage is that in preparing these MSP nanodisks, one relies on the use of surfactants hindering the study of membrane proteins in their native lipid environment. Besides, the use of peptides as rim-stabilizing molecules complicates the use of biophysical techniques such as circular dichroism, Fourier transform infrared and NMR spectroscopies (9).

An alternative approach to MSP is the use of amphipathic copolymers. These copolymers keep membrane proteins soluble without detergents (9–14). This implies that membrane proteins, together with their annular lipid shells, can be extracted directly from native cellular membranes or from reconstituted vesicles. An efficient copolymer introduced by Dafforn and coworkers (9,10) is composed of styrene-maleic acid (SMA) units. SMA molecules, together with lipids, spontaneously form disk-shaped particles of 10–12 nm in diameter, which are denoted as SMA lipid particles (SMALPs) (15). Bigger particles may also form depending on the shape and diameter of the embedded protein(s), polymer composition, and the polymer/lipid ratio (10,16–19). Importantly, SMA copolymers dissolve in a wide range of membranes without showing specificity for any lipid types (20–22). They have been used to characterize

Submitted February 2, 2018, and accepted for publication June 11, 2018.
Correspondence s.j.marrink@rug.nl
Editor: Alemayehu Gorfe.
https://doi.org/10.1016/j.bpj.2018.06.018
© 2018 Biophysical Society.
This is an open access article under the CC BY license (http://creativecommons.org/licenses/by/4.0/).

the annular lipid shells of a variety of membrane proteins (23–25). The intrinsic hydrophobicity (SMA ratio) and the protonation state of maleic acid groups strongly influence the rate of membrane solubilization (26,27). These properties, together with the varying molecular weight, make SMA copolymers easy to change and adjust (28,29). As a result, these pH-responsive copolymers have also been used as membrane-destabilizing polymers for the delivery of therapeutic molecules (26,30).

Despite the promising future of SMA copolymers in membrane protein research, little is known about the molecular mechanism of SMA-lipid nanodisk formation. Scheidelaar and colleagues suggested a model for membrane solubilization by SMA copolymers in which the hydrophobic effect would drive the interaction between SMA copolymers and membranes, modulated by electrostatic interactions (20). Molecular dynamics (MD) simulations provide an attractive tool to study the molecular interactions and the dynamics of the solubilization process in detail (31). Considering the large molecular weight of the polymers, coarse-grained (CG) models are required to access the relatively large timescales involved in membrane destabilization (32–35). A CG model that has been parametrized for both polymeric systems and lipid membranes is the Martini model (36). This model has already been successfully applied to simulate the interaction of a variety of polymers and lipid membranes, including studies on polymer adsorption (37–43), polymer-mediated fusion (44), the permeation process of dendrimers (45) and polymer-coated nanoparticles (46,47), and preformed lipid nanodisks (48–53).

Here, based on CG MD simulations with the Martini model, we describe the molecular mechanism of action of SMA copolymers in destabilizing a model lipid bilayer. We provide detailed insight into the insertion, penetration, and pore formation of these copolymers and show how they cooperatively lead to complete destabilization of the lipid membrane and the onset of nanodisk formation. Besides, self-assembly experiments of SMA copolymers and lipids of different length show that small nanodisks are the preferred end state.

METHODS

CG SMA model

The Martini CG model is used for the parametrization of the basic SMA units (54). Herein, we used SMA copolymers consisting of 23 units, with each unit including two styrene groups and one maleic acid, yielding a molecular weight of ~7.4 kDa, which is similar to the molecular weight used in previous experiments (9,13,27). The copolymers were treated as fully deprotonated with two negative charges in each repeating unit to obtain the high aqueous solubility of these copolymers and to avoid aggregation. For the styrene group, a three-bead mapping scheme was used, similar to the ring-based side chains in the existing Martini models for the aromatic phenylalanine and tyrosine amino acids and the styrene group in the polystyrene molecule (55,56). For the maleic acid groups, a one-bead representation was used to represent the carboxylic group, carrying a full negative charge each. The chosen mapping of the CG SMA copolymer is shown in Fig. 1 *a*.

Bonds and improper dihedral angles were represented based on standard harmonic potentials, whereas angles and proper dihedral angles were modeled with cosine-based potentials and periodic dihedral potentials, respectively. The set of CG bonded parameters was parametrized by comparison with atomistic simulations of the SMA copolymers at the interface between an aqueous solution and dodecane. Constraints were applied to the aromatic CG beads instead of using ordinary bonds. The target distribution functions were obtained for the various bonds, angles, and dihedrals from the atomistic trajectory. In a couple of iterative steps, the CG parameters were adjusted to obtain the best match between the pseudo-CG and real-CG distributions. A full description of the CG topology and a comparison with atomistic data can be found in Figs. S1, S2, and Table S1. The SMA model is available at http://cgmartini.nl.

Simulation details

We used the Martini 2.2P force field to model the interactions between lipid membranes and SMA copolymers (54,57,58). The primary setup consists of a bilayer composed of 1352 didecanoylphosphatidylcholine (DDPC) lipids built using the INSANE script (59) and 1, 10, or 20 SMA copolymers

a

b

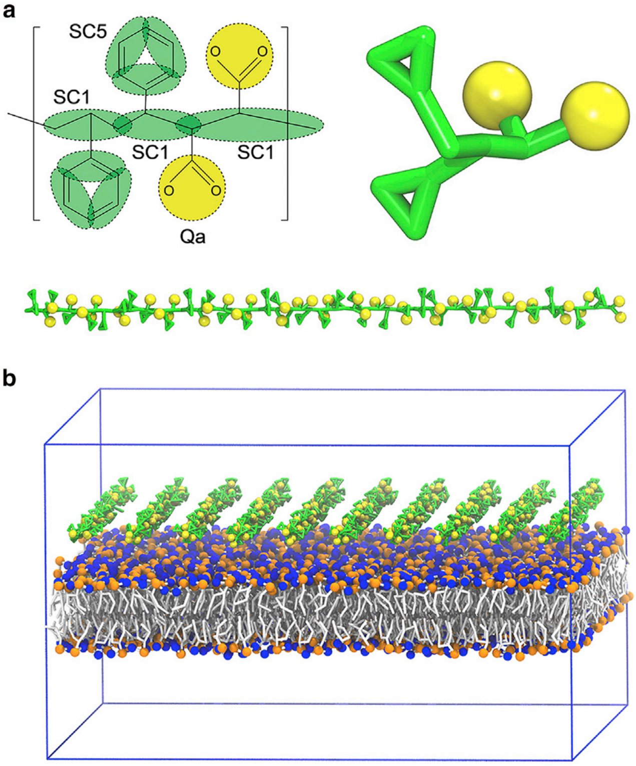

FIGURE 1 SMA model and starting configuration of the simulation. (*a*) A CG model for the SMA copolymer with mapping of the CG SMA model and chosen bead types is shown at the top left, and a zoomed-in image of one CG unit is shown at the top right. (*b*) The initial configuration of the simulation system with 10 SMA copolymers above the preformed DDPC lipid bilayer is shown. DDPC lipids are shown in gray with phosphate groups in orange and choline groups in blue. The SMA copolymers are shown in green, and the carboxyl groups are shown in yellow. The periodic boundary condition box is shown with blue solid lines. The solvent is omitted for clarity. To see this figure in color, go online.

regularly arranged at a distance of 2.0 nm away from the lipid surface (Fig. 1 *b*). The solvent layer in those systems comprised between 24,527 and 38,526 water beads, wherein one bead represented four real water molecules. We used a concentration of 150 mM of sodium chloride, which is optimal for nanodisk formation according to previous experimental works (20,27). All systems were neutralized by adding extra sodium ions. After minimization, all systems were first equilibrated at constant volume (NVT) and then at constant pressure (NPT, with semi-isotropic coupling) at a temperature of 310 K, using a Berendsen barostat and a V-rescale thermostat (60,61). After equilibration, we changed the barostat to the Parrinello-Rahman method (62) while the standard Martini water model was still used, which proved to be more efficient at initiating the insertion of the polymers to the membrane surface (within a few hundred nanoseconds). We also applied a flat-bottomed potential on the copolymers to keep them close to the membrane solvent interface. The harmonic distance restraints keep the copolymers within a distance of 3.0 nm around the membrane surface, and the potential was released once the copolymers attached to the membrane. At this point, the standard water model was replaced by the polarizable Martini water model (63) to mimic the electrostatic interactions more realistically.

For each polymer concentration, we performed between two and five replicas starting from random initial velocities. Most simulations reached up to 3 μs. To assess the thermodynamic stability of the nanodisks, self-assembly simulations were performed, starting from a random mixture of all the components, using 4, 8, or 16 SMA polymers with 600 lipids (corresponding to 150:1, 75:1, and 75:2 lipid ratios) and excess water. A polarizable water model was used, and two replicas were performed for each polymer/lipid ratio. All simulations were run using Groningen Machine for Chemical Simulations version 5.0.7 (64). The total simulation time covered over 60 μs. To test the effect of polymer charge and lipid tail type, additional simulations were performed using 50% instead of fully charged polymers and longer-tail DMPC (dimyristoyl-PC), DPPC (dipalmitoyl-PC), or polyunsaturated dilinoleoyl-PC lipids. An overview of all simulations is provided in Table S2. Details of the atomistic simulations performed to calibrate the CG interactions can be found in the Supporting Materials and Methods.

RESULTS AND DISCUSSION

SMA copolymers spontaneously inserted into the lipid bilayer

The starting setup of our simulations consisted of a bilayer composed of 1352 DDPC lipids. The short tail DDPC should facilitate membrane disruption on the accessible timescale of our simulations. We placed 1, 10, or 20 SMA copolymers in the aqueous phase in the vicinity of one of the membrane leaflets (Fig. 1 *b*). The initial asymmetric placement of the copolymers represents the experimental situation in which polymers are added in the solution surrounding a liposome or cell. Each SMA polymer consisted of 23 monomeric units and was fully deprotonated with two negative charges per monomer. The highly charged state mimics conditions of high pH, guaranteeing a highly soluble state of the polymers (27,65), although the experimentally highest efficiency is obtained at somewhat lower pH (27). We performed multiple runs for each condition to increase the statistics (Table S2). In all cases, the SMA copolymers quickly adopted a disordered conformation in solution (Fig. 2 *a*), in agreement with potentiometric studies and with all-atom simulations (Fig. S3) (66). In the case of the simulation system with 10 or 20 SMA copolymers, the copolymers could also self-aggregate through their hydrophobic cores in solution, as shown experimentally (27).

The membrane affinity of the SMA copolymers, however, is high. We observed the spontaneous insertion of SMA copolymers already in the early phases (10–500 ns) of

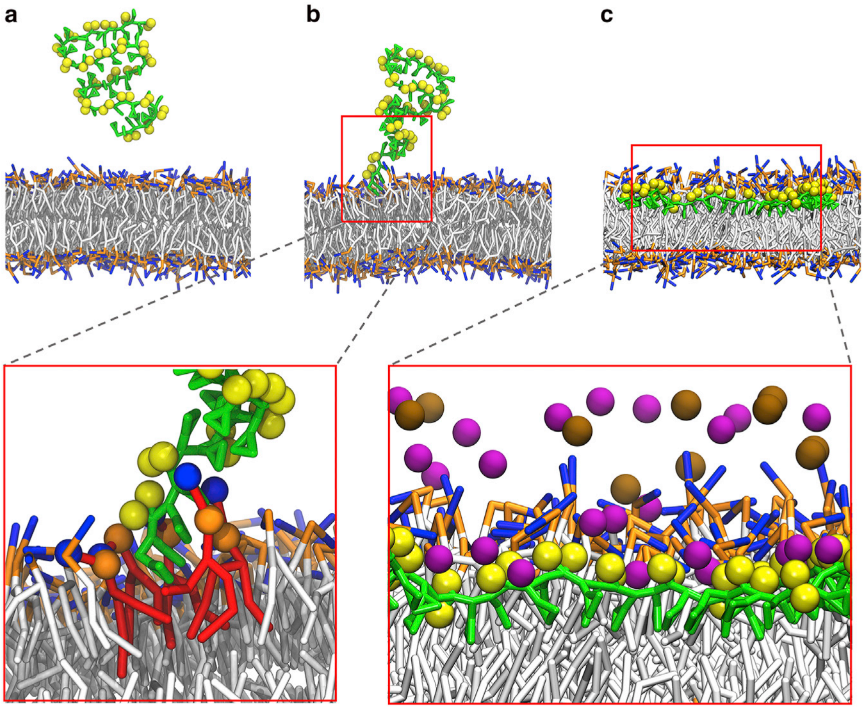

FIGURE 2 Snapshots of the binding process of an SMA copolymer to the surface of the membrane. (*a*) At the beginning ($t = 0$ ns), the polymer is in solution, taking a disordered conformation. (*b*) After 20 ns, the polymer adheres on the surface of the membrane through the hydrophobic terminal inserted between the lipid acyl tails. (*c*) At $t = 400$ ns, the polymer is fully absorbed to the lipid bilayer with sodium ions mediating the electrostatic interactions between the SMA carboxyl and the lipid headgroups. Styrene groups are shown in green, and carboxyl groups are shown in yellow. Lipids are shown with gray tails, orange phosphate, and blue choline groups. Lipids around the hydrophobic termini are highlighted in red in the left insert, with blue and orange beads representing the choline and phosphate groups, respectively. Purple beads represent Na^+ ions, and brown beads represent Cl^- ions in the right insert. Some lipids in front of the polymer as well as water molecules were removed for clarity. Snapshots were obtained for a system with one copolymer. To see this figure in color, go online.

most of our simulations. Molecular insertion always started with the styrene moieties of the SMA polymeric termini (Fig. 2 *b*; Video S1). Hydrophobic interactions between the styrene moieties of the polymers and the lipid acyl chains seem to drive this behavior. The termini appeared to be strongly bound to the lipids because detachments were not observed after insertion. Once this is achieved, the rest of the copolymer slowly followed. This increases the interaction between the copolymer and the water-lipid interface. The inserted copolymers were located under the phosphate headgroups with the styrene moieties fitting between the acyl chains and the carboxyl groups pointing to the solution (Fig. 2 *c*). In the adsorbed state, the polymers became stretched. The analysis of the radius of gyration confirmed this change in structure upon membrane binding (Fig. S4). Counterions seem to play also an important role in stabilizing the polymer-lipid interactions (Fig. 2 *c*, *inset*). Analysis of the density profiles along the membrane normal revealed an asymmetric distribution of sodium ions around the membrane (Fig. S5 *a*). Insertion of SMA copolymers dragged additional sodium ions into the lipid/water interface. This seems to help the copolymers to overcome the repulsion between the charged carboxyl groups and the lipid phosphate groups (Fig. S5, *b* and *c*).

SMA copolymers perturbed the bilayer, inducing pore formation

The binding of SMA copolymers induced cooperative activities that included membrane bending, lipid extraction, lipid tilting, and water infiltration. In particular, when multiple polymers aggregated, the insertion of the SMA copolymers produced significant local bending of the membrane around the insertion site (Fig. 3 *a*). The bending originated from the increased size of the hydrophobic core in the leaflet to which they absorbed, causing stress and distorting the planarity of the lipid bilayer. In some simulations, the aggregate pulled lipids out of the membrane, ending up in the hydrophobic core of the polymers in solution (Fig. 3 *b*). This, however, might be facilitated by the short tail length of the lipids used and become more difficult with typical phospholipids. In most of our simulations, penetration of the copolymers caused infiltration of water molecules between the lipids' tails. This made the lipids close to the copolymers tilt and shield their tails from the carboxyl groups of the copolymers and the water molecules. Some lipids even toppled over, lying horizontal to the membrane surface (Fig. 3 *c*). The disorder in the lipid bilayer allowed other water molecules on the other side to cross the lipid bilayer, forming transmembrane pores. At the same time, the polymers bridged to the other side, spanning across the membrane (Fig. 3 *c*). Together, the lipid flip-flopping and polymer translocation relieved the stress imbalance induced by the asymmetric adsorption of the SMA copolymers. Again, the timescale of this process is likely dependent on the length of the lipid tail and artificially enhanced in our simulations.

SMA stabilized the pore rim, resulting in pore growth and membrane disruption

Even though the initial stress imbalance largely dissipated, water permeation increased after the initial transmembrane

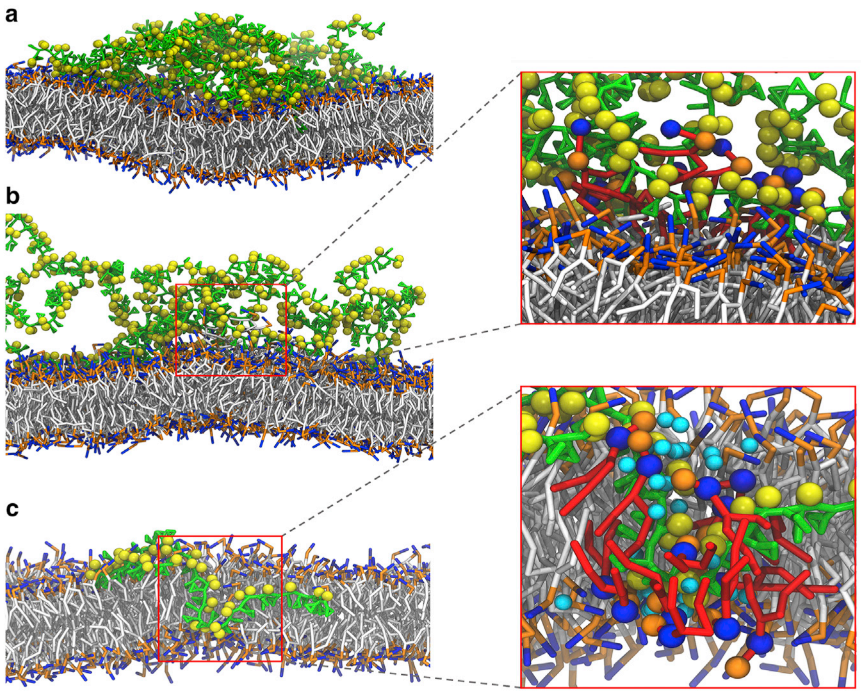

FIGURE 3 Membrane perturbations induced by SMA copolymers. (*a*) Membrane bending induced by the increased size of the hydrophobic core of the polymer cluster. (*b*) Lipids were pulled off from the membrane into the hydrophobic core of the polymer cluster. Extracted lipids are highlighted in red in the zoomed-in view. (*c*) Permeation of water molecules is shown close to an SMA copolymer (only one polymer chain shown). Colors are the same as in Figs. 1 and 2. Lipids around the water pore are highlighted in red in the zoomed-in view. Water molecules involved in the permeation through the membrane are depicted in cyan. In all cases, snapshots were obtained from independent simulations of a system containing 10 copolymers. To see this figure in color, go online.

pores formed. The amphipathic nature of SMA copolymers would likely favor the interaction with the water molecules inside the pore and the lipid tails. This further stabilized the pore's rim. The hydrophobic styrene groups intercalated perpendicularly to the lipid acyl chains, which agrees with the polarized attenuated total reflection Fourier-transform infrared spectroscopy measurements (9). The SMA carboxyl groups and the nearby lipid headgroups faced toward the water pore. This forced the lipids around the pore to tilt, forming a toroidal pore. At the beginning, the pores showed a roughly cylindrical shape and became more irregular as the pores expanded (Fig. 4 *a*). At the end of the simulations with high concentration of SMA copolymers, we observed big pores forming (with diameters of 5–10 nm) and the original bilayer largely destroyed (see Fig. 4 *b*). At this point, the systems seemed to reach a metastable state, preventing the complete formation of nanodisks. It is possible that the periodic boundary conditions used in the simulation artificially stabilized the connectivity in the plane of the membrane, resulting in a kinetic trap. The formation of the full pore upon SMA copolymer binding is shown in Video S2. We also quantified the kinetics of pore expansion by measuring the sizes of several pores over time (Fig. S6 *c*).

Complete SMALP nanodisks formed by self-assembly

To test the capability of SMA copolymers to form stable SMALPs and to avoid metastable states in preformed membrane bilayers, we also performed self-assembly experiments. We used a mixture of SMA copolymers and DDPC lipids (either 4, 8, or 16 SMA copolymer molecules per 600 lipid molecules), which corresponds to 150:1, 75:1, or 75:2 lipid/polymer ratios. Without copolymers, lipids form stable bilayers in self-assembly simulations (57). However, when we added SMA copolymers to DDPC lipids, stable SMALPs formed with the SMA copolymer bound to the edge of the lipid bilayer disk (Fig. 5). Often a few micelles initially also remained present. As the simulations progressed, however, these micelles merged with other nanodisks through the exposed polymer-depleted sides. The

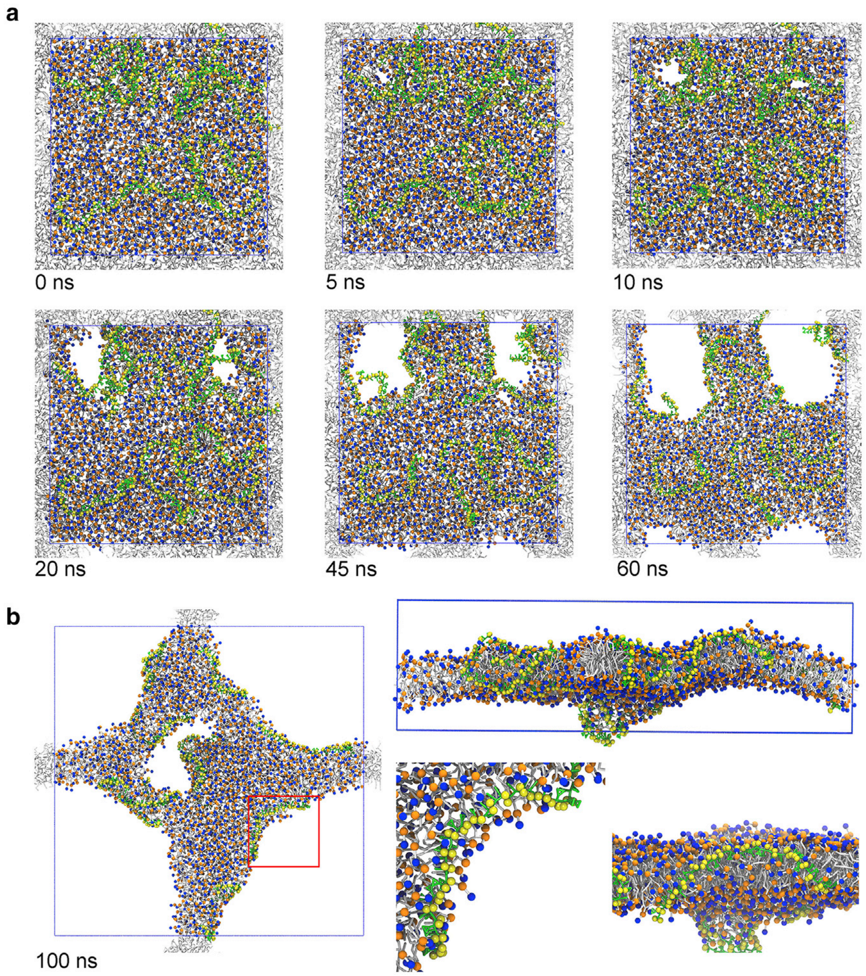

FIGURE 4 Pore formation upon SMA copolymer binding to the lipid bilayer. (*a*) A top view of the process shows first the SMA binding (t = 0 ns) and the initial water permeation (t = 5 ns), followed by the pore formation (t = 10 ns) and growth of those pores in the membrane (t = 20–60 ns) for a system containing 10 copolymers. The simulation times are shown in each figure, with the time of the first snapshot reset to zero. (*b*) The final snapshot of the simulation (t = 100 ns) is shown from both top and side views. The pore edge is shown in more detail at the bottom right (*top view* and *side view*). SMA copolymers are shown in green and yellow. Lipids are depicted with gray tails and orange/blue spheres for phosphate/choline groups. Water molecules are removed for clearness. Periodic boundary boxes are shown as blue grid lines. Lipids in other periodic boxes are shown in gray. To see this figure in color, go online.

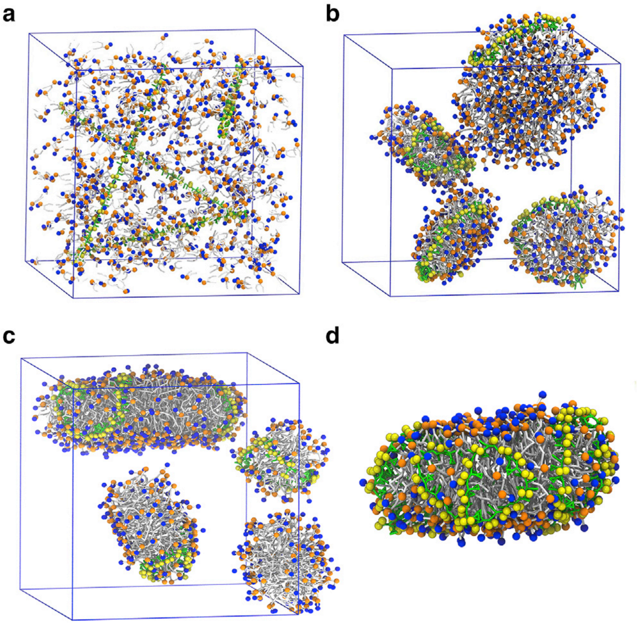

FIGURE 5 Formation of SMALPs in the self-assembly experiment. (*a*) The initial random distribution of DDPC lipids and SMA copolymers is shown at a 150:1 SMA/lipid ratio. (*b*) The resulting SMALP nanodisks are shown. (*c*) SMALP nanodisk and micelles are formed in self-assembly simulation using DPPC lipids. (*d*) A snapshot is shown of a single nanodisk surrounded with multiple copolymers, formed in SMA and DPPC self-assembly simulation at a 75:2 SMA/lipid ratio. The same coloring is used as in the other figures. To see this figure in color, go online.

formed nanodisks were always stable. The embedding of the SMA copolymers during the self-assembly simulations was similar to what we observed during the membrane-disruption simulations. SMA copolymers stabilized the pore rim with the styrene and the carboxyl groups in opposite directions (Fig. 4 *b*).

In the self-assembly simulations with a 150:1 lipid/polymer ratio, the SMA copolymers formed four nanodisks, with one polymer chain per nanodisk. The diameters of the nanodisks ranged from 7 to 9 nm. This agrees with the overall structural measurements of free SMALPs in solution. Small-angle neutron scattering measurements have shown that the inner radius of SMA nanodisks is around 3.8 ± 0.2 nm (10). The nanodisks comprised one polymer in their annulus, in line with experimental data showing nanodisks surrounded by a one polymer thick belt (9). Despite the use of a different lipid composition in those experiments, earlier experiments have shown that the particle shape and the average diameter of the nanodisks are independent of the acyl-chain length (20).

To test whether SMA copolymers can form nanodisks with long-tail lipids, we performed additional self-assembly simulations, replacing DDPC with either DMPC (myristoyl chains, 14 carbons) or DPPC (palmitoyl chains, 16 carbons) lipids. In both cases, stable nanodisks formed with similar sizes and lipid/copolymer ratios compared to DDPC (Fig. S7). With DPPC, however, we often observed two SMA polymer chains per nanodisk, which can be explained by the larger size of the hydrophobic core (Fig. 5 *c*). To further investigate the polymer concentration effect on the nanodisks formed, we also performed self-assembly simulations at higher (75:1 and 75:2 lipid/polymer ratio) polymer concentrations. Again, nanodisks formed (Fig. S7), but several SMA copolymers surrounded the lipid disk, in agreement with the general idea that multiple polymer chains are required to completely surround a nanodisk (67). Fig. 5 *d* shows an example of such a nanodisk.

Limitations of our model and further controls

Simulations of membrane solubilization at an all-atom level of resolution are computationally too expensive and cannot currently be performed. The use of a CG model allows such computations but implies that some detail is lost. An extensive discussion of the assumptions and limitations underlying the Martini model can be found in (36). Here, we briefly discuss the main limitations that could influence our results. One such limitation is the directionality of hydrogen bonds, which is missing in the Martini model; hydrogen bonds are represented isotropically only. Previous work, however, indicates that this is not an important limitation in capturing the essence of membrane-polymer interactions (37–52). Another limitation, in particular when compared to experimental settings, is the small system sizes considered in this study combined with periodic boundary conditions. The latter may cause an artificial stabilization of the lamellar phase, which makes it more difficult for the polymers to break the membrane into nanodisks. Together with the limited timescales that can be reached by our simulations (microsecond range), this prompted us to select the short-tail DDPC lipids to speed up the process. Energy barriers for pore formation in longer-tail lipids are large, estimated to be around 45 and 78 kJ/mol for DMPC and DPPC, respectively, according to previous all-atom simulations (68). Pore formation is even harder to observe in Martini CG simulations (69). Additional simulations using longer-tail lipids, DMPC or DPPC, and the polyunsaturated dilinoleoyl-PC lipids revealed that the initial adsorption process of the polymers was similar to what we observed for DDPC lipids (Fig. S8), but pores did not form. Therefore, pore formation probably requires longer timescales. However, we expect the mechanism of SMALP formation to be generic. Reassuringly, we observed similar nanodisks forming upon self-assembly, comparing short-tail lipids to more common longer-tail lipids, as shown in Figs. 5 and S7. This data suggests that rupture of intact membranes of longer-tail lipids eventually will also happen with our models but perhaps requires the use of smart sampling techniques to observe the process of pore formation.

Another important difference regarding typical experimental settings is the optimal polymer charge density and

lipid/polymer ratio. To probe the effect of polymer charge, we performed additional simulations using 50% charged SMA copolymers (i.e., every second maleic acid unit was considered protonated). Experimental data suggest that dissociation of ~50% is more appropriate (27). Our simulations showed that changing the charged state of the polymer did not lead to qualitative differences for those conditions tested (see Fig. S6). One difference, though, was that the pores expanded at different rates, with the fully charged model expanding faster than the half-protonated one (Fig. S6, *c* and *d*), probably due to the larger charge density inside the pore in the case of the fully charged model. Concerning the lipid/polymer ratio, the experiments show that nanodisk formation is more efficient at lipid/polymer weight ratios of between 1:1 and 1:3, depending on the type of copolymers (16). However, control simulations with higher polymer concentrations (up to ~3:1 lipid/polymer weight ratio) led to extensive clustering of the polymers in the aqueous phase (Fig. S9). This clustering behavior severely hampered the adsorption and insertion efficiency of the polymers into the membrane. On an experimental timescale, this is no problem, but on our simulation timescale, it is. Fortunately, using the self-assembly setup, it was possible to explore higher polymer concentrations, revealing nanodisk formation at a 75:2 lipid/polymer molar ratio (~3:2 weight ratio), with multiple polymers stabilizing the rim (Fig. 5 *d*).

CONCLUSIONS

We investigated the molecular mechanism of the early stages of SMA nanodisk formation using CG MD simulations. Despite the limitations associated with our model, we expect our findings to be generic. More detailed all-atom models should validate our results. Based on our simulations, we propose the following mechanism for SMA-induced nanodisk formation:

1. SMA copolymers bind to the membrane surface through the styrene moieties of the termini. The hydrophobic interactions drive the initial insertion with the core of the lipid bilayer.
2. Full insertion of the SMA copolymers' hydrophobic side chains follows, causing local membrane undulation.
3. Translocation of the SMA copolymers relieves the induced stress, together with water molecules and accommodated by lipid flip-flop. Small transmembrane pores form.
4. Growth of the transmembrane pores occurs. The SMA copolymers stabilize the rim by orienting the carboxyl moieties to the water pore, and the benzene groups intercalated in between the lipid tails. This likely disrupts the membrane and favors nanodisk formation. Because of periodic boundary effects, we could not observe the last phase, but self-assembly simulations show that SMALP nanodisks are the thermodynamically favorable state of the system.

Our findings show the solubilization ability of SMA copolymers and the details of the process at the molecular level. Our simulation protocol paves the way for further studies of SMA nanodisks, exploring different conditions (pH, polymer composition, and multicomponent lipid membranes) and will help the design of optimized copolymers for nanodisk formation and drug-delivery systems. Simulations of nanodisk formation with membrane-embedded proteins are underway. They will contribute to understanding the influence of SMA copolymers on the structural and dynamic properties of proteins and their annular lipid shells in SMA nanodisks.

SUPPORTING MATERIAL

Supporting Materials and Methods, nine figures, two tables, and two videos are available at http://www.biophysj.org/biophysj/supplemental/S0006-3495(18)30721-5.

AUTHOR CONTRIBUTIONS

M.X., I.F., and S.J.M. designed the research. M.X. and L.C. performed the research. M.X. and I.F. analyzed the data. M.X., I.F., W.G., and S.J.M. wrote the article.

ACKNOWLEDGMENTS

We would like to thank Antoinette Killian, Stefan Scheidelaar, and Martijn Koorengevel for many helpful discussions.

This work was supported by the China Scholarship Council, 973 Program (2013CB932604), National Natural Science Foundation of China (51535005 and 51472117), the Research Fund of State Key Laboratory of Mechanics and Control of Mechanical Structures (MCMS-0416K01 and MCMS-0416G01), the Fundamental Research Funds for the Central Universities (NP2017101), a project funded by the Priority Academic Program Development of Jiangsu Higher Education Institutions, and the funding of the Jiangsu Innovation Program for Graduate Education (KYLX_0225). The laboratory of S.J.M. is supported by a European Research Council advanced grant COMP-MICR-CROW-MEM.

REFERENCES

1. Engel, A., and H. E. Gaub. 2008. Structure and mechanics of membrane proteins. *Annu. Rev. Biochem.* 77:127–148.
2. Lee, S. C., S. Khalid, ..., T. R. Dafforn. 2016. Encapsulated membrane proteins: a simplified system for molecular simulation. *Biochim. Biophys. Acta.* 1858:2549–2557.
3. Marty, M. T., K. K. Hoi, and C. V. Robinson. 2016. Interfacing membrane mimetics with mass spectrometry. *Acc. Chem. Res.* 49:2459–2467.
4. Inagaki, S., R. Ghirlando, and R. Grisshammer. 2013. Biophysical characterization of membrane proteins in nanodiscs. *Methods.* 59:287–300.
5. Denisov, I. G., and S. G. Sligar. 2017. Nanodiscs in membrane biochemistry and biophysics. *Chem. Rev.* 117:4669–4713.
6. Rouck, J. E., J. E. Krapf, ..., A. Das. 2017. Recent advances in nanodisc technology for membrane protein studies (2012-2017). *FEBS Lett.* 591:2057–2088.

7. Bayburt, T. H., Y. V. Grinkova, and S. G. Sligar. 2002. Self-assembly of discoidal phospholipid bilayer nanoparticles with membrane scaffold proteins. *Nano Lett.* 2:853–856.
8. Denisov, I. G., and S. G. Sligar. 2016. Nanodiscs for structural and functional studies of membrane proteins. *Nat. Struct. Mol. Biol.* 23:481–486.
9. Jamshad, M., V. Grimard, …, T. R. Dafforn. 2014. Structural analysis of a nanoparticle containing a lipid bilayer used for detergent-free extraction of membrane proteins. *Nano Res.* 8:774–789.
10. Knowles, T. J., R. Finka, …, M. Overduin. 2009. Membrane proteins solubilized intact in lipid containing nanoparticles bounded by styrene maleic acid copolymer. *J. Am. Chem. Soc.* 131:7484–7485.
11. Long, A. R., C. C. O'Brien, …, N. N. Alder. 2013. A detergent-free strategy for the reconstitution of active enzyme complexes from native biological membranes into nanoscale discs. *BMC Biotechnol.* 13:41.
12. Gulati, S., M. Jamshad, …, A. J. Rothnie. 2014. Detergent-free purification of ABC (ATP-binding-cassette) transporters. *Biochem. J.* 461:269–278.
13. Dörr, J. M., M. C. Koorengevel, …, J. A. Killian. 2014. Detergent-free isolation, characterization, and functional reconstitution of a tetrameric K+ channel: the power of native nanodiscs. *Proc. Natl. Acad. Sci. USA.* 111:18607–18612.
14. Popot, J. L. 2010. Amphipols, nanodiscs, and fluorinated surfactants: three nonconventional approaches to studying membrane proteins in aqueous solutions. *Annu. Rev. Biochem.* 79:737–775.
15. Lee, S. C., T. J. Knowles, …, T. R. Dafforn. 2016. A method for detergent-free isolation of membrane proteins in their local lipid environment. *Nat. Protoc.* 11:1149–1162.
16. Orwick, M. C., P. J. Judge, …, A. Watts. 2012. Detergent-free formation and physicochemical characterization of nanosized lipid-polymer complexes: Lipodisq. *Angew. Chem. Int. Ed. Engl.* 51:4653–4657.
17. Paulin, S., M. Jamshad, …, P. W. Taylor. 2014. Surfactant-free purification of membrane protein complexes from bacteria: application to the staphylococcal penicillin-binding protein complex PBP2/PBP2a. *Nanotechnology.* 25:285101.
18. Zhang, R., I. D. Sahu, …, G. A. Lorigan. 2015. Characterizing the structure of lipodisq nanoparticles for membrane protein spectroscopic studies. *Biochim. Biophys. Acta.* 1848:329–333.
19. Ravula, T., S. K. Ramadugu, …, A. Ramamoorthy. 2017. Bioinspired, size-tunable self-assembly of polymer-lipid bilayer nanodiscs. *Angew. Chem. Int. Ed. Engl.* 56:11466–11470.
20. Scheidelaar, S., M. C. Koorengevel, …, J. A. Killian. 2015. Molecular model for the solubilization of membranes into nanodisks by styrene maleic acid copolymers. *Biophys. J.* 108:279–290.
21. Cuevas Arenas, R., J. Klingler, …, S. Keller. 2016. Influence of lipid bilayer properties on nanodisc formation mediated by styrene/maleic acid copolymers. *Nanoscale.* 8:15016–15026.
22. Dominguez Pardo, J. J., J. M. Dörr, …, J. A. Killian. 2017. Solubilization of lipids and lipid phases by the styrene-maleic acid copolymer. *Eur. Biophys. J.* 46:91–101.
23. Prabudiansyah, I., I. Kusters, …, A. J. Driessen. 2015. Characterization of the annular lipid shell of the Sec translocon. *Biochim. Biophys. Acta.* 1848:2050–2056.
24. Swainsbury, D. J., S. Scheidelaar, …, M. R. Jones. 2014. Bacterial reaction centers purified with styrene maleic acid copolymer retain native membrane functional properties and display enhanced stability. *Angew. Chem. Int. Ed. Engl.* 53:11803–11807.
25. Rehan, S., V. O. Paavilainen, and V. P. Jaakola. 2017. Functional reconstitution of human equilibrative nucleoside transporter-1 into styrene maleic acid co-polymer lipid particles. *Biochim. Biophys. Acta.* 1859:1059–1065.
26. Henry, S. M., M. E. El-Sayed, …, P. S. Stayton. 2006. pH-responsive poly(styrene-alt-maleic anhydride) alkylamide copolymers for intracellular drug delivery. *Biomacromolecules.* 7:2407–2414.
27. Scheidelaar, S., M. C. Koorengevel, …, J. A. Killian. 2016. Effect of polymer composition and pH on membrane solubilization by styrene-maleic acid copolymers. *Biophys. J.* 111:1974–1986.
28. Oluwole, A. O., B. Danielczak, …, S. Keller. 2017. Solubilization of membrane proteins into functional lipid-bilayer nanodiscs using a diisobutylene/maleic acid copolymer. *Angew. Chem. Int. Ed. Engl.* 56:1919–1924.
29. Yasuhara, K., J. Arakida, …, A. Ramamoorthy. 2017. Spontaneous lipid nanodisc fomation by amphiphilic polymethacrylate copolymers. *J. Am. Chem. Soc.* 139:18657–18663.
30. Banerjee, S. S., N. Aher, …, J. Khandare. 2012. Poly(ethylene glycol)-prodrug conjugates: concept, design, and applications. *J. Drug Deliv.* 2012:103973.
31. Ingólfsson, H. I., C. Arnarez, …, S. J. Marrink. 2016. Computational 'microscopy' of cellular membranes. *J. Cell Sci.* 129:257–268.
32. Ingólfsson, H. I., C. A. Lopez, …, S. J. Marrink. 2014. The power of coarse graining in biomolecular simulations. *Wiley Interdiscip. Rev. Comput. Mol. Sci.* 4:225–248.
33. Chavent, M., A. L. Duncan, and M. S. Sansom. 2016. Molecular dynamics simulations of membrane proteins and their interactions: from nanoscale to mesoscale. *Curr. Opin. Struct. Biol.* 40:8–16.
34. Pluhackova, K., and R. A. Böckmann. 2015. Biomembranes in atomistic and coarse-grained simulations. *J. Phys. Condens. Matter.* 27:323103.
35. Saunders, M. G., and G. A. Voth. 2013. Coarse-graining methods for computational biology. *Annu. Rev. Biophys.* 42:73–93.
36. Marrink, S. J., and D. P. Tieleman. 2013. Perspective on the Martini model. *Chem. Soc. Rev.* 42:6801–6822.
37. Hezaveh, S., S. Samanta, …, D. Roccatano. 2012. Understanding the interaction of block copolymers with DMPC lipid bilayer using coarse-grained molecular dynamics simulations. *J. Phys. Chem. B.* 116:14333–14345.
38. Pizzirusso, A., A. De Nicola, and G. Milano. 2016. MARTINI coarse-grained model of triton TX-100 in pure DPPC monolayer and bilayer interfaces. *J. Phys. Chem. B.* 120:3821–3832.
39. Adhikari, U., A. Goliaei, …, M. L. Berkowitz. 2016. Properties of poloxamer molecules and poloxamer micelles dissolved in water and next to lipid bilayers: results from computer simulations. *J. Phys. Chem. B.* 120:5823–5830.
40. Barnoud, J., G. Rossi, …, L. Monticelli. 2014. Hydrophobic compounds reshape membrane domains. *PLoS Comput. Biol.* 10:e1003873.
41. Rossi, G., J. Barnoud, and L. Monticelli. 2014. Polystyrene nanoparticles perturb lipid membranes. *J. Phys. Chem. Lett.* 5:241–246.
42. Bochicchio, D., E. Panizon, …, G. Rossi. 2017. Interaction of hydrophobic polymers with model lipid bilayers. *Sci. Rep.* 7:6357.
43. Wood, I., M. F. Martini, …, M. Pickholz. 2016. Coarse grained study of pluronic F127: comparison with shorter co-polymers in its interaction with lipid bilayers and self-aggregation in water. *J. Mol. Struct.* 1109:106–113.
44. Pannuzzo, M., D. H. De Jong, …, S. J. Marrink. 2014. Simulation of polyethylene glycol and calcium-mediated membrane fusion. *J. Chem. Phys.* 140:124905.
45. Lee, H., and R. G. Larson. 2008. Coarse-grained molecular dynamics studies of the concentration and size dependence of fifth- and seventh-generation PAMAM dendrimers on pore formation in DMPC bilayer. *J. Phys. Chem. B.* 112:7778–7784.
46. Oroskar, P. A., C. J. Jameson, and S. Murad. 2016. Simulated permeation and characterization of PEGylated gold nanoparticles in a lipid bilayer system. *Langmuir.* 32:7541–7555.
47. Lee, H. 2013. Interparticle dispersion, membrane curvature, and penetration induced by single-walled carbon nanotubes wrapped with lipids and PEGylated lipids. *J. Phys. Chem. B.* 117:1337–1344.
48. Perlmutter, J. D., W. J. Drasler, II, …, J. N. Sachs. 2011. All-atom and coarse-grained molecular dynamics simulations of a membrane protein stabilizing polymer. *Langmuir.* 27:10523–10537.

49. Shih, A. Y., P. L. Freddolino, …, K. Schulten. 2007. Assembly of lipoprotein particles revealed by coarse-grained molecular dynamics simulations. *J. Struct. Biol.* 157:579–592.

50. Vuorela, T., A. Catte, …, I. Vattulainen. 2010. Role of lipids in spheroidal high density lipoproteins. *PLoS Comput. Biol.* 6:e1000964.

51. Ollila, O. H., A. Lamberg, …, I. Vattulainen. 2012. Interfacial tension and surface pressure of high density lipoprotein, low density lipoprotein, and related lipid droplets. *Biophys. J.* 103:1236–1244.

52. Popovic, K., J. Holyoake, …, G. G. Privé. 2012. Structure of saposin A lipoprotein discs. *Proc. Natl. Acad. Sci. USA.* 109:2908–2912.

53. Debnath, A., and L. V. Schäfer. 2015. Structure and dynamics of phospholipid nanodiscs from all-atom and coarse-grained simulations. *J. Phys. Chem. B.* 119:6991–7002.

54. Marrink, S. J., H. J. Risselada, …, A. H. de Vries. 2007. The MARTINI force field: coarse grained model for biomolecular simulations. *J. Phys. Chem. B.* 111:7812–7824.

55. Monticelli, L., S. K. Kandasamy, …, S. J. Marrink. 2008. The MARTINI coarse-grained force field: extension to proteins. *J. Chem. Theory Comput.* 4:819–834.

56. Rossi, G., L. Monticelli, …, T. Ala-Nissila. 2011. Coarse-graining polymers with the MARTINI force-field: polystyrene as a benchmark case. *Soft Matter.* 7:698–708.

57. Marrink, S. J., A. H. de Vries, and A. E. Mark. 2004. Coarse grained model for semiquantitative lipid simulations. *J. Phys. Chem. B.* 108:750–760.

58. de Jong, D. H., S. Baoukina, …, S. J. Marrink. 2016. Martini straight: boosting performance using a shorter cutoff and GPUs. *Comput. Phys. Commun.* 199:1–7.

59. Wassenaar, T. A., H. I. Ingólfsson, …, S. J. Marrink. 2015. Computational lipidomics with insane: a versatile tool for generating custom membranes for molecular simulations. *J. Chem. Theory Comput.* 11:2144–2155.

60. Berendsen, H. J. C., J. P. M. Postma, …, J. R. Haak. 1984. Molecular dynamics with coupling to an external bath. *J. Chem. Phys.* 81:3684–3690.

61. Bussi, G., D. Donadio, and M. Parrinello. 2007. Canonical sampling through velocity rescaling. *J. Chem. Phys.* 126:014101.

62. Parrinello, M., and A. Rahman. 1981. Polymorphic transitions in single crystals: a new molecular dynamics method. *J. Appl. Phys.* 52:7182–7190.

63. Yesylevskyy, S. O., L. V. Schäfer, …, S. J. Marrink. 2010. Polarizable water model for the coarse-grained MARTINI force field. *PLoS Comput. Biol.* 6:e1000810.

64. Abraham, M. J., T. Murtola, …, E. Lindahl. 2015. GROMACS: high performance molecular simulations through multi-level parallelism from laptops to supercomputers. *SoftwareX.* 1–2:19–25.

65. Banerjee, S., T. K. Pal, and S. K. Guha. 2012. Probing molecular interactions of poly(styrene-co-maleic acid) with lipid matrix models to interpret the therapeutic potential of the co-polymer. *Biochim. Biophys. Acta.* 1818:537–550.

66. Tonge, S. R., and B. J. Tighe. 2001. Responsive hydrophobically associating polymers: a review of structure and properties. *Adv. Drug Deliv. Rev.* 53:109–122.

67. Dörr, J. M., S. Scheidelaar, …, J. A. Killian. 2016. The styrene-maleic acid copolymer: a versatile tool in membrane research. *Eur. Biophys. J.* 45:3–21.

68. Bennett, W. F., N. Sapay, and D. P. Tieleman. 2014. Atomistic simulations of pore formation and closure in lipid bilayers. *Biophys. J.* 106:210–219.

69. Bennett, W. F., and D. P. Tieleman. 2011. Water defect and pore formation in atomistic and coarse-grained lipid membranes: pushing the limits of coarse graining. *J. Chem. Theory Comput.* 7:2981–2988.

Biophysical Journal, Volume *115*

Supplemental Information

Molecular Mechanism of Lipid Nanodisk Formation by Styrene-Maleic Acid Copolymers

Minmin Xue, Lisheng Cheng, Ignacio Faustino, Wanlin Guo, and Siewert J. Marrink

Supplementary Information

Molecular Mechanism of Lipid Nanodisc Formation by Styrene Maleic Acid Copolymers

Minmin Xue[1,2,3], Lisheng Cheng[4], Ignacio Faustino[2,3], Wanlin Guo[1], and Siewert J. Marrink*[2,3].

[1]State Key Laboratory of Mechanics and Control of Mechanical Structures and Key Laboratory for Intelligent Nano Materials and Devices of the Ministry of Education, and Institute of Nanoscience, Nanjing University of Aeronautics and Astronautics, Nanjing 210016, People's Republic of China.

[2]Groningen Biomolecular Science and Biotechnology Institute, University of Groningen, Nijenborgh 7, 9747 AG, Groningen, The Netherlands.

[3]Zernike Institute for Advanced Materials, University of Groningen, Nijenborgh 4, 9747 AG, Groningen, The Netherlands.

[4]College of Mechanical and Electrical Engineering, Beijing University of Chemical Technology, Beijing 100029, People's Republic of China

SUPPLEMENTARY METHODS

Parametrization and validation of the Martini SMA model

Quantum mechanics calculations: The chirality sequence in short SMA polymers has an effect on the conformational properties of the linear polymer[1]. Based on preliminary atomistic simulations of short SMA chains with different chirality, we noticed that the bonded distributions showed better convergence using a tetramer. To predict the most stable stereo isomeric tetramers, we used quantum mechanics calculations for all possible stereoisomers (2^4) (see Fig. S1). We used a short chain of styrene and maleic anhydride copolymer since this is the precursor before hydrolysis during the industrial production of the SMA-2000 copolymer. We used the semi-empirical PM6 method because it accounts for accurate intramolecular interactions and has the advantage of being less time-consuming than other DFT methods. The conformational search of each tetramer was performed in the gas phase at the PM6 level of theory. Eventually, the five most stable chirality sequences were used for the parametrization of the coarse-grained (CG) SMA model.

Mapping: The mapping of the CG beads was chosen based on the mapping of previous Martini models (see Fig. 1). For the styrene group, a three-beads mapping scheme was used similar to the ring-based side chains in the aromatic phenylalanine and tyrosine amino acids and the styrene group in the Martini polystyrene model. For the maleic acid group, a one-bead representation was used to represent the carboxylic group, carrying a full negative charge each. The full SMA model is available at http://cgmartini.nl.

Bonded and non-bonded parameters of the Martini SMA model: Biphasic simulations were used for the parametrization of the Martini SMA model. Based on the semi-empirical quantum mechanics calculations, we built five different SMA copolymers based on the most stable SMA tetramers (see Table S1). In these simulations, one SMA copolymer was placed at the interface of water and dodecane. The system size was set to 4.4×4.4×6.9 nm^3 and a similar number of water and dodecane molecules were included. Sodium ions were added to neutralize the negative charge of the SMA copolymer. Bonds, angles and proper and improper dihedral angles of the CG molecules were optimized by comparison with atomistic simulations using the OPLS-AA force field[2]. The details of the CG simulations can be found in the *Methods* section of the main manuscript. The target distribution functions were obtained after a few iterative steps and were agree remarkably well with the atomistic distributions (Fig. S2a). We tested the CG models by calculating the radii of gyration depending on polymer chain length, and by comparing the results obtained from CG and AA simulations (Fig. S2b). A very good agreement between the CG and AA models was obtained.

SMA copolymers in solutions: To further validate the CG model, we considered the behavior of the full SMA copolymer chain in solutions. Here we compared our CG model with an atomistic model using the CHARMM36 force parameters set[3]. The system consists of a single SMA chain of 23 monomeric units in excess aqueous solvent. Constant temperature was maintained at 310 K and constant pressure was maintained at 1 atm. Full electrostatic forces were evaluated using the particle-mesh Ewald method with a cutoff of 1.4 nm[4]. Short non-bonded terms were evaluated every step using a cutoff of 1.4 nm for van der Waals interactions. The last 200 ns of the simulation were used for data analysis, with the first 50 ns considered as equilibrium. Previous potentiometric titration studies of SMA copolymers showed that, at neutral and high pH, the electrostatic repulsions between the carboxylic groups dominate the hydrophobic interactions between styrene groups within the polymers, resulting in a disordered conformation that dissolves relatively easily in aqueous solution[5, 6]. Both our all-atom and CG simulations showed that the fully charged SMA copolymer adopts a mostly disordered conformation in agreement with the potentiometric studies (Fig. S3a,b). The conformations adopted by the polymers range from partly collapsed to full extended. Visual inspection indicates also the presence of loops forming within one copolymer, providing some shielding of the styrene moieties (see Fig. 2A in the main manuscript). Both atomistic and CG MD simulations of the SMA copolymer showed similar flexibility properties according to the end-to-end distances and radius of gyration (Fig. S3c and S3d).

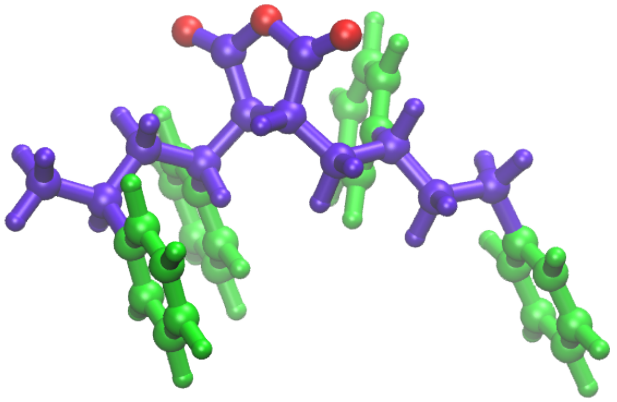

Figure S1 | The structure of a short chain of styrene-maleic anhydride copolymer used to calculate the configurational energy with quantum mechanics.

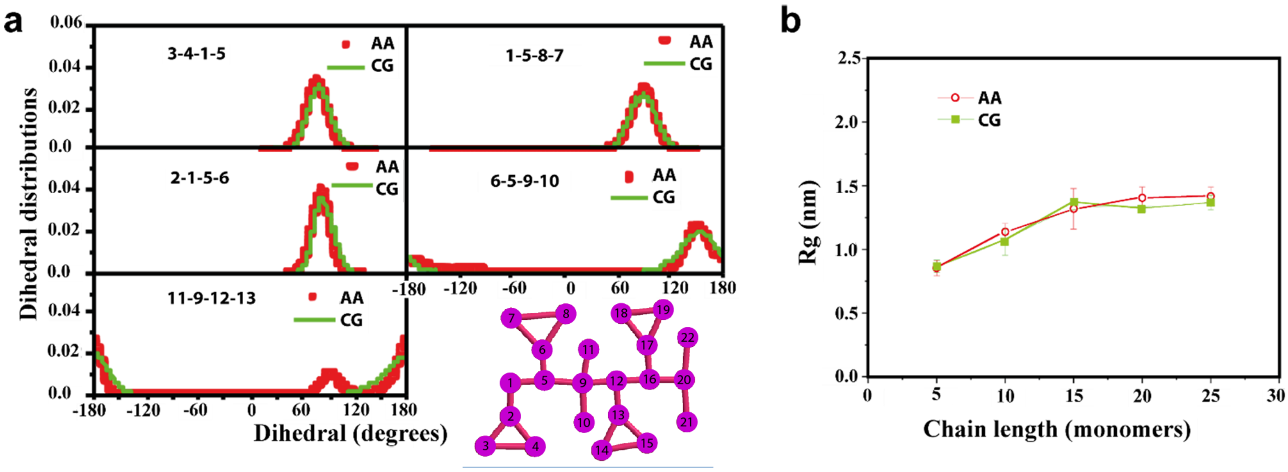

Figure S2 | Analysis of the Martini SMA model and comparison with OPLS-AA atomistic simulations. (**a**) Distributions of dihedral angles obtained from atomistic simulations and optimized coarse grain parameters. The numbers correspond to the beads in CG topology. (**b**) Radius of gyration depending on the polymer chain length. The copolymer is composed of the five monomer types in a random order.

Chirality sequence	Configurational energy (kJ/mol)
RRRR	-0.53
RRRL*	-10.85
RRLR*	-14.31
RRLL	3.37
RLRR	21.44
RLRL	8.98
RLLR	0.79
RLLL	21.44
LRRR	-1.05
LRRL*	-13.34
LRLR*	-17.94
LRLL*	-11.55
LLRR	10.0
LLRL	2.64
LLLR	-1.84
LLLL	2.68

Supplementary Table S1 | Energies of the sixteen chirality sequences of optimized geometries of *styrene-styrene-maleic acid-maleic acid* tetramers. Configurational energies were calculated using the PM6 semi-empirical method. (*) Configurations corresponding to the lowest configurational energies among the 16 combinations. L and R denote the left and right chiralities of the SMA units, respectively.

Type of simulation	Lipid type	Copolymer type (percentage charged)	Number of SMA copolymers/lipids	Number of replicas	Simulation length (us)	Number of pores (bilayer) / nanodiscs (self-assembly) formed
Bilayer	DDPC	100%	1 / 1352	2	2.4	0 / 0
			10 / 1352	5	3.0	2 / 3 / 3 / 2 / 3
			10 / 1352	3	0.5	0 / 1 / 0
			20 / 1352	2	0.4	1 / 0
			40 / 1352	2	0.2	0 / 0
		50%	10 / 1352	2	1.0	3 / 4
	DMPC	100%	1 / 1352	2	1.0	0 / 0
			10 / 1352	2	2.0	0 / 0
			20 / 1352	1	1.0	0
		50%	10 / 1352	1	1.0	0
	DPPC	100%	1 / 1352	2	1.0	0 / 0
			10 / 1352	2	2.0	0 / 0
		50%	10 / 1352	1	1.0	0
	DIPC	100%	10 / 1352	2	3.0	0 / 0
			20 / 1352	2	2.0	0 / 0
		50%	10 / 1352	1	1.0	0
Self-assembly	DDPC	100%	4 / 600	2	2.0	4 / 4
			8 / 600	2	0.9	5 / 7
			16 / 600	2	1.0	11 / 12
		50%	16 / 600	1	1.0	9
	DMPC	100%	4 / 600	2	2.0	4 / 3
			8 / 600	2	1.0	1 / 3
			16 / 600	2	1.0	7 / 8
		50%	16 / 600	1	1.0	8
	DPPC	100%	4 / 600	2	2.0	4 / 3
			8 / 600	2	1.0	4 / 5
			16 / 600	2	1.0	6 / 7
		50%	16 / 600	1	1.0	7

Table S2. Details and outcomes for bilayer and self-assembly simulations used in this work. Only bilayer simulations with regularly placed SMA copolymers are listed here.

SUPPLEMENTARY RESULTS

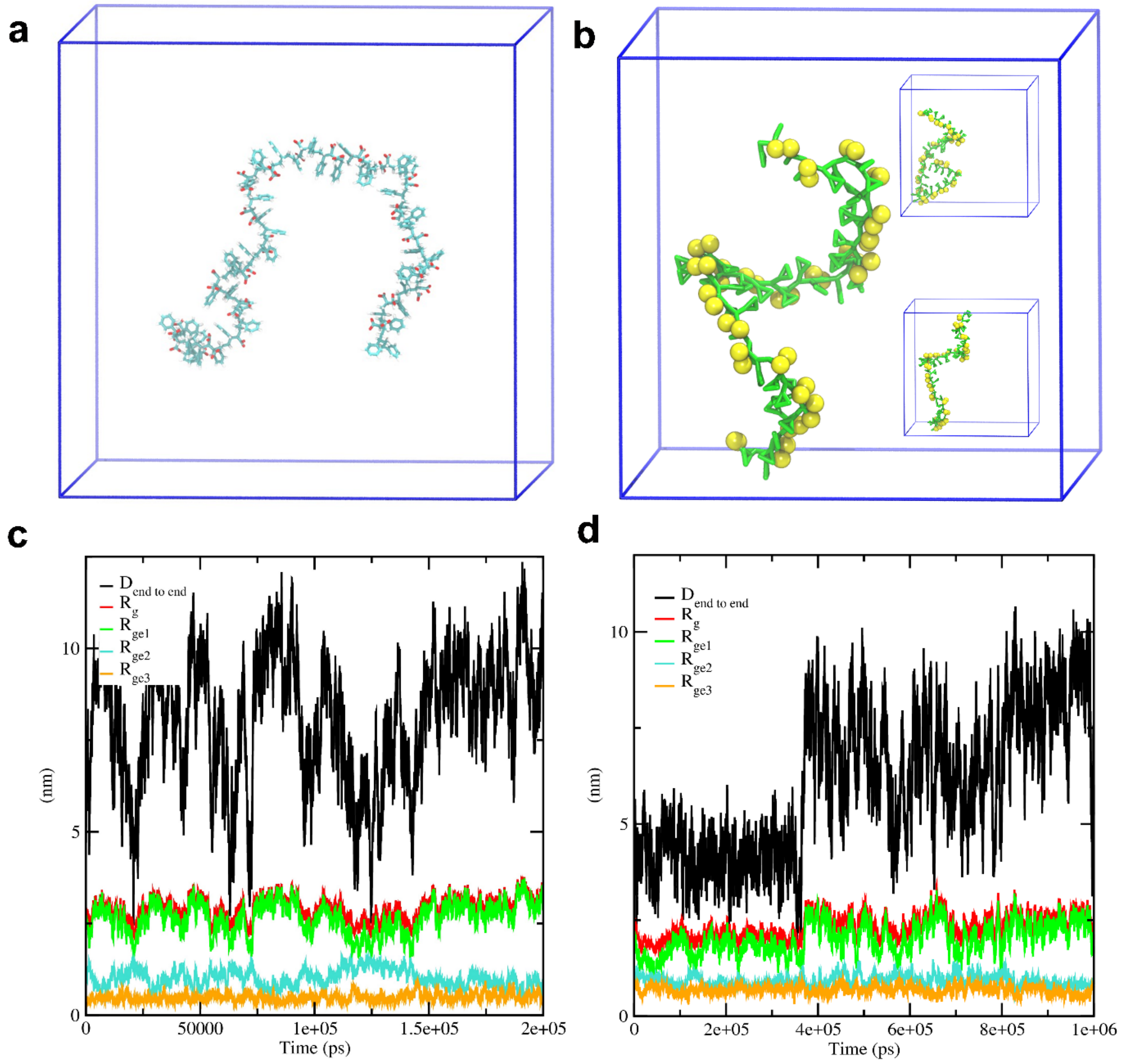

Figure S3 | Comparison of the polymer flexibility properties obtained from atomistic (left) and coarse grain (right) simulations of SMA copolymers in aqueous solvent. In the upper part, a schematic view of SMA copolymers in (**a**) all-atom and (**b**) coarse-grained representations is shown. The bottom row shows the time evolution of the end-to-end distances and radii of gyration in (**c**) atomistic and (**d**) coarse-grained MD simulations. The jump in the end-to-end distance in (**d**) is due to a conformational change from a partially collapsed to a fully stretched out conformation, as is shown in the insets in (**b**).

Effect of SMA copolymer adsorption on membrane properties and polymer end-to-end distance

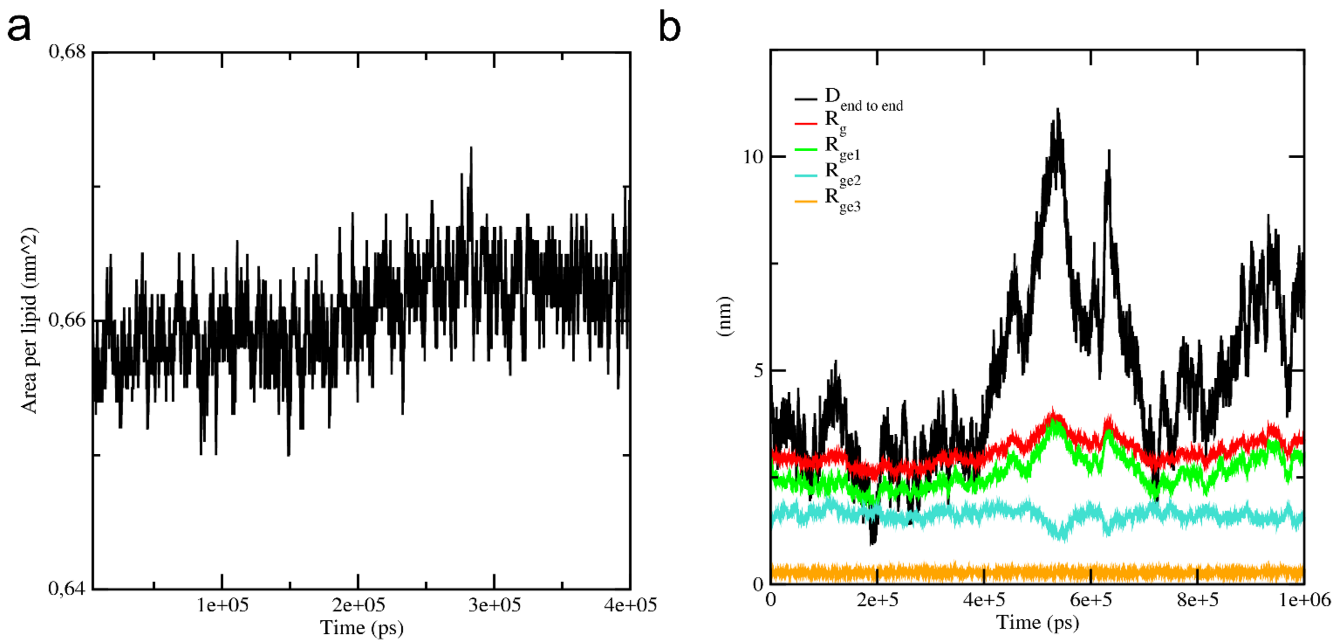

Figure S4 | Analysis of some properties of the membrane and the SMA copolymer during the attachment process. (**a**) The increase in the area per lipid is due to the binding and lying down of the SMA copolymer on the surface of membrane. (**b**) Change in the end-to-end distance and radius of gyration of the SMA copolymer. The results correspond to one of the simulations with 1 SMA copolymer. Similar results were obtained in other simulations at high SMA concentration.

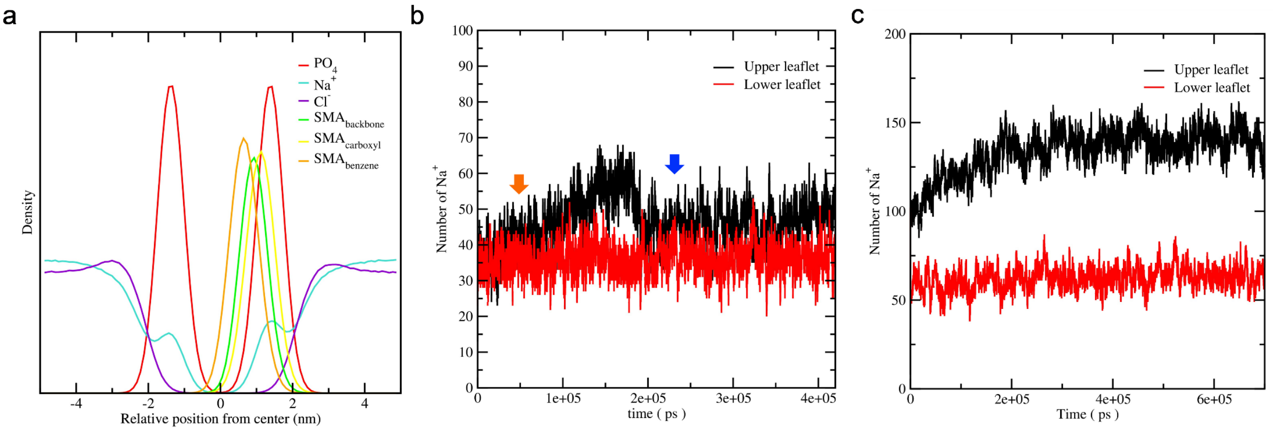

Figure S5 | Density profiles across the z-axis and sodium ion distribution in the lipid/water interface. (**a**) Density profiles across the z-axis of the membrane after the SMA copolymer is attached in CG simulations. The benzene rings insert deeper into the hydrophobic core while the carboxyl groups point towards the solvent. The results correspond to one of the simulations with 1 SMA copolymer. Similar results were obtained in the other simulations at high SMA concentration. (**b**) Time evolution of number of Na^+ ions within 7 Å of the phosphate head group on both sides of the DTPC membrane in the simulation with 1 SMA

copolymer on top. The orange and blue arrows indicate the time the SMA copolymer starts to insert into the membrane and becomes fully stretched out on the surface, respectively. (c) Time evolution of number of Na^+ ions upon adsorption on both sides of the DTPC membrane in system with 10 SMA copolymers on top of the upper leaflet.

Simulations of half charged SMA copolymers

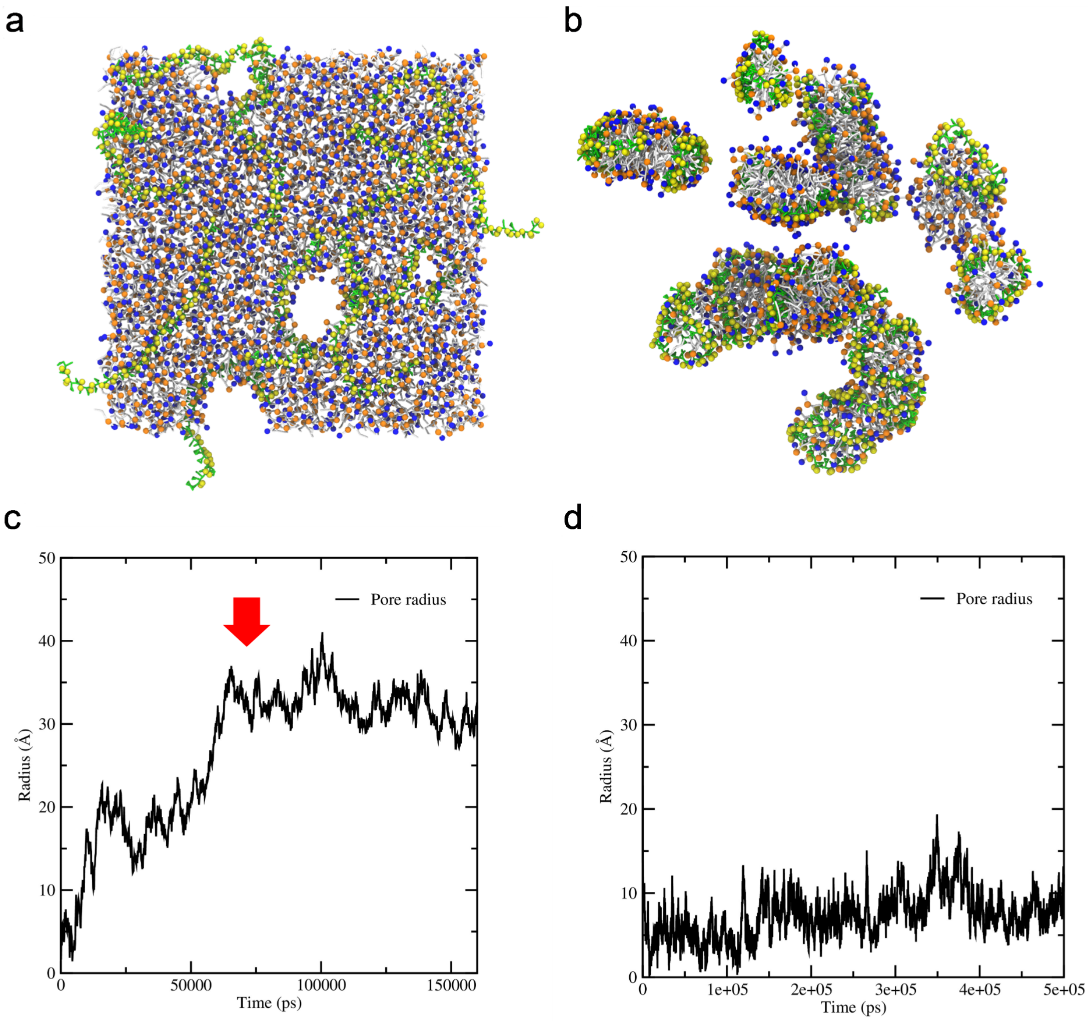

Figure S6 | Simulation results of half charged copolymers. (**a**) Snapshot of the simulation using 10 SMA copolymers and DDPC bilayer at 500 ns. (**b**) Self-assembly simulation of DDPC SMALPs using 2:75 polymer-to-lipid ratio. (**c**) Radius for one of the pores in the disrupted DDPC bilayer from fully charged model. The pore stops growing until trapped in a metastable state (red arrow). (**d**) Radius for one of the pores in the disrupted DDPC bilayer from half charged model.

Self-assembly process for nanodiscs of DDPC, DMPC and DPPC lipids

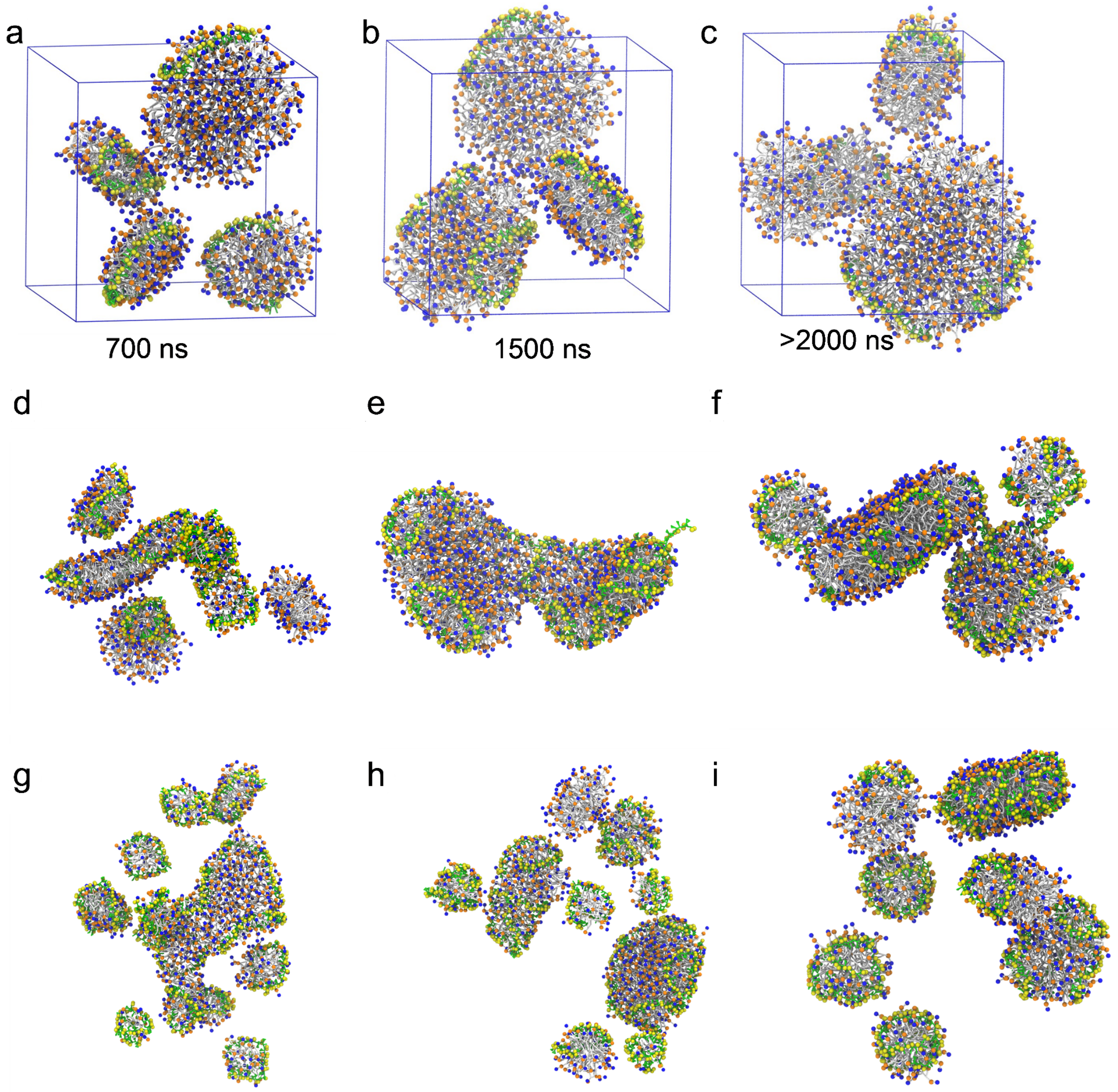

Figure S7 | Formation of SMALPs during self-assembly experiments with DDPC, DMPC and DPPC lipids using CG simulations. (**a**) DDPC SMALPs, (**b**) DMPC SMALPs and (**c**) DPPC SMALPs assemblies after 2 microsecond simulation with polymer-to-lipid ratio of 1:150. PBC boxes are shown in blue grid lines. Simulation time required for nanodiscs self-assembly are shown for each system. (**d**) DDPC SMALPs, (**e**) DMPC SMALPS and (**f**) DPPC SMALPs assemblies after 1 microsecond simulation with polymer-to-lipid ratio of 1:75. (**g**) DDPC SMALPs, (**h**) DMPC SMALPs and (**i**) DPPC SMALPs assemblies after 1 microsecond simulation with polymer-to-lipid ratio of 2:75.

Adsorption of SMA copolymers on top of long tail lipid membrane surface

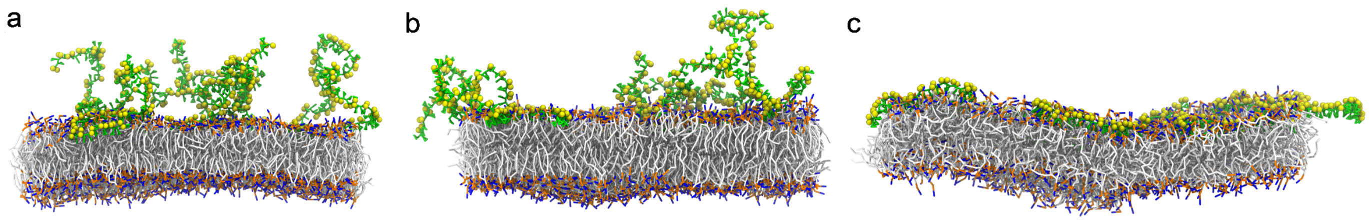

Figure S8 | Snapshots of adsorption of 10 SMA copolymers on DMPC, DPPC and DLiPC membrane surface after 2 us simulation. (**a**) Adsorption of 10 SMA on DMPC membrane. (**b**) Adsorption of 10 SMA on DPPC membrane. (**c**) Adsorption of 10 SMA on DLiPC membrane. Solvents are omitted for clarity. Some copolymers are fully adsorbed while some still have part of it in solution. Faster and complete adsorption of SMA copolymers are observed for DLiPC membrane, which indicates DLiPC could be a potential candidate for pore formation in nanodisc computational research.

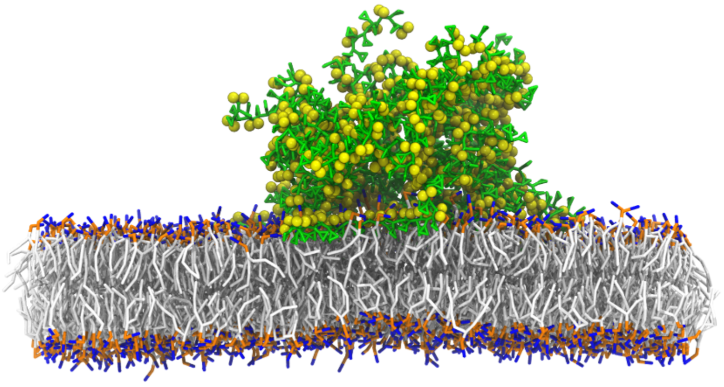

Figure S9 | Snapshots of adsorption of 20 SMA copolymer on DMPC membrane surface. Cluster of aggregated 20 SMA were formed and adsorbed on surface of DMPC membrane after 1 µs simulation. Solvents are omitted for clarity.

Supporting Citations

1. Malardier-Jugroot C, van de Ven TGM, Whitehead MA. Linear conformation of poly (styrene-*alt*-maleic anhydride) capable of self-assembly: a result of chain stiffening by internal hydrogen bonds. *The Journal of Physical Chemistry B* **109**, 7022-7032 (2005).

2. Jorgensen WL, Maxwell DS, Tirado-Rives J. Development and Testing of the OPLS All-Atom Force Field on Conformational Energetics and Properties of Organic Liquids. *Journal of the American Chemical Society* **118**, 11225-11236 (1996).

3. Vanommeslaeghe K*, et al.* CHARMM general force field: A force field for drug-like molecules compatible with the CHARMM all-atom additive biological force fields. *Journal of computational chemistry* **31**, 671-690 (2010).

4. Essmann U, Perera L, Berkowitz ML, Darden T, Lee H, Pedersen LG. A smooth particle mesh Ewald method. *The Journal of Chemical Physics* **103**, 8577-8593 (1995).

5. Ohno N, Nitta K, Makino S, Sugai S. Conformational transition of the copolymer of maleic acid and styrene in aqueous solution. *Journal of Polymer Science Part A-2: Polymer Physics* **11**, 413-425 (1973).

6. Tonge SR, Tighe BJ. Responsive hydrophobically associating polymers: a review of structure and properties. *Advanced Drug Delivery Reviews* **53**, 109-122 (2001).
